# Atomic view into *Plasmodium* actin polymerization, ATP hydrolysis, and fragmentation

**DOI:** 10.1371/journal.pbio.3000315

**Published:** 2019-06-14

**Authors:** Esa-Pekka Kumpula, Andrea J. Lopez, Leila Tajedin, Huijong Han, Inari Kursula

**Affiliations:** 1 Biocenter Oulu and Faculty of Biochemistry and Molecular Medicine, University of Oulu, Oulu, Finland; 2 Department of Biomedicine, University of Bergen, Bergen, Norway; 3 European XFEL GmbH, Schenefeld, Germany; The Beatson Institute, UNITED KINGDOM

## Abstract

*Plasmodium* actins form very short filaments and have a noncanonical link between ATP hydrolysis and polymerization. Long filaments are detrimental to the parasites, but the structural factors constraining *Plasmodium* microfilament lengths have remained unknown. Using high-resolution crystallography, we show that magnesium binding causes a slight flattening of the *Plasmodium* actin I monomer, and subsequent phosphate release results in a more twisted conformation. Thus, the Mg-bound monomer is closer in conformation to filamentous (F) actin than the Ca form, and this likely facilitates polymerization. A coordinated potassium ion resides in the active site during hydrolysis and leaves together with the phosphate, a process governed by the position of the Arg178/Asp180-containing A loop. Asp180 interacts with either Lys270 or His74, depending on the protonation state of the histidine, while Arg178 links the inner and outer domains (ID and OD) of the actin protomer. Hence, the A loop acts as a switch between stable and unstable filament conformations, the latter leading to fragmentation. Our data provide a comprehensive model for polymerization, ATP hydrolysis and phosphate release, and fragmentation of parasite microfilaments. Similar mechanisms may well exist in canonical actins, although fragmentation is much less favorable due to several subtle sequence differences as well as the methylation of His73, which is absent on the corresponding His74 in *Plasmodium* actin I.

## Abstract 2

A detailed mechanistic study of malaria parasite actins reveals at the atomic level how they polymerize, hydrolyze ATP, and are fragmented to keep actin filament lengths short enough for parasite survival.

## Introduction

Actin is the constituent protein of microfilaments with essential roles in central processes in the cell, including transport, cell division, and motility [1–3]. The primary biological activity of actin is its polymerization to form filaments that can generate force at cell membranes or act as scaffolding structures or tracks for motor proteins [4]. These filaments are on a timer, based on the hydrolysis of tightly bound ATP, formation of the stable intermediate ADP-inorganic phosphate (P_i_) actin, and finally, the release of P_i_ [5]. The physiological tightly bound divalent cation coordinating the bound nucleotide in actin is Mg^2+^ (reviewed in [6]). However, actin is usually purified in the calcium-bound form, which has a higher critical concentration of polymerization [7]. In addition to magnesium and calcium, at least potassium affects actin polymerization by decreasing the critical concentration by approximately 2-fold [7].

In model actins, which typically represent actins of opisthokonts from yeast to mammals, the coupling of nucleotide hydrolysis to filament stability is well established. In general, ADP actin depolymerizes much faster than ATP or ADP-P_i_ actin and is therefore the main depolymerizing species [7]. Although ADP actin can polymerize, its critical concentration is much higher than that of ATP actin [7], which leads to domination of ATP actin in polymerization kinetics. Outliers of this functional consensus are actins of the phylum Apicomplexa, including *Plasmodium* spp. and *Toxoplasma gondii*—both notorious human pathogens. With less than 80% sequence identity to their canonical counterparts, actins of these parasites are among the most evolutionarily diverged eukaryotic actins while still retaining most of the core features [8–11]. The primary actin of *P*. *falciparum* and the only one of *T*. *gondii* are the best understood of the phylum, whereas others remain virtually uncharacterized.

In vitro, apicomplexan actins tend to form only short filaments of approximately100 nm without the filament-stabilizing macrolide jasplakinolide [8–10,12]. *T*. *gondii* actin (*Tg*Act) has been proposed to follow an isodesmic polymerization mechanism [11], which would differ fundamentally from the classical nucleation-elongation pathway. However, *P*. *falciparum* actin I (*Pf*ActI) has a critical concentration close to that of mammalian α-actin and a very similar elongation rate [13]. Under ADP-rich conditions, *Pf*ActI forms oligomers of 3 to 12 subunits while forming larger polymeric species in polymerizing conditions containing magnesium and potassium, together with a significant pool of dimers [9,13]. These properties are in stark contrast to what is seen for the well-characterized model systems.

Despite the functional differences, the *Pf*ActI monomer largely resembles canonical actins in structure [9]. The most pronounced differences are at the pointed end, namely, subdomain (SD) 2 (containing the DNaseI-binding D loop) and parts of SD4, which both connect to SD3 of the next longitudinal protomer in the filament. The D loop and the C terminus are both important functional factors but are disordered in the crystal structure of *Pf*ActI, reflecting their flexibility [9]. In jasplakinolide-stabilized *Pf*ActI filaments, the D loop is in a clearly altered conformation compared with α-actin filaments [10]. Yet the main hydrophobic interactions are conserved, and the amino acid substitutions are primarily located at the base of the D loop [9]. In addition, differences in the plug region (residues Ser266-Ala273, especially Lys270) and some other residues along the filament interface (in particular Val288, Gly200) also likely contribute to filament instability [10]. However, a single key factor driving the inherent instability of the parasite microfilaments has not been identified.

The large-scale conformational transition of the actin monomer from globular (G) to filamentous (F) form has been described from a series of high-resolution filament structures of α-actin [14–18]. Yet experimental evidence on what exactly triggers the structural transition and the subsequent activation of ATP hydrolysis is still lacking. Among key questions are the following: (i) Why does Mg-ATP actin polymerize more readily than Ca-ATP actin or Mg-ADP actin? (ii) What is the role of K^+^ in polymerization and ATP hydrolysis? Unlike the extensively studied model actins, *Pf*ActI forms short oligomers also in classical nonpolymerizing conditions in the presence of ADP and, on the other hand, stable dimers—in addition to short filamentous structures—in conditions in which canonical actins polymerize into long filaments [9,13]. Thus, it seems that hydrolysis of ATP and subsequent P_i_ release is favorable for oligomerization of *Pf*ActI. Associated structural changes could thus favor nucleus formation—i.e., result in a conformation closer to the F than the G state. Here, we analyze phosphate release rates and high-resolution structures of wild-type and mutant *Plasmodium* actins in different nucleotide states, bridging the gap between structure and function in understanding the polymerization mechanism and the instability of the parasite microfilaments. Many of the conclusions may be relevant for understanding these mechanisms also in canonical actins.

## Results

### Unusual coupling between nucleotide hydrolysis and polymerization in *Pf*ActI

**Fig 1:**
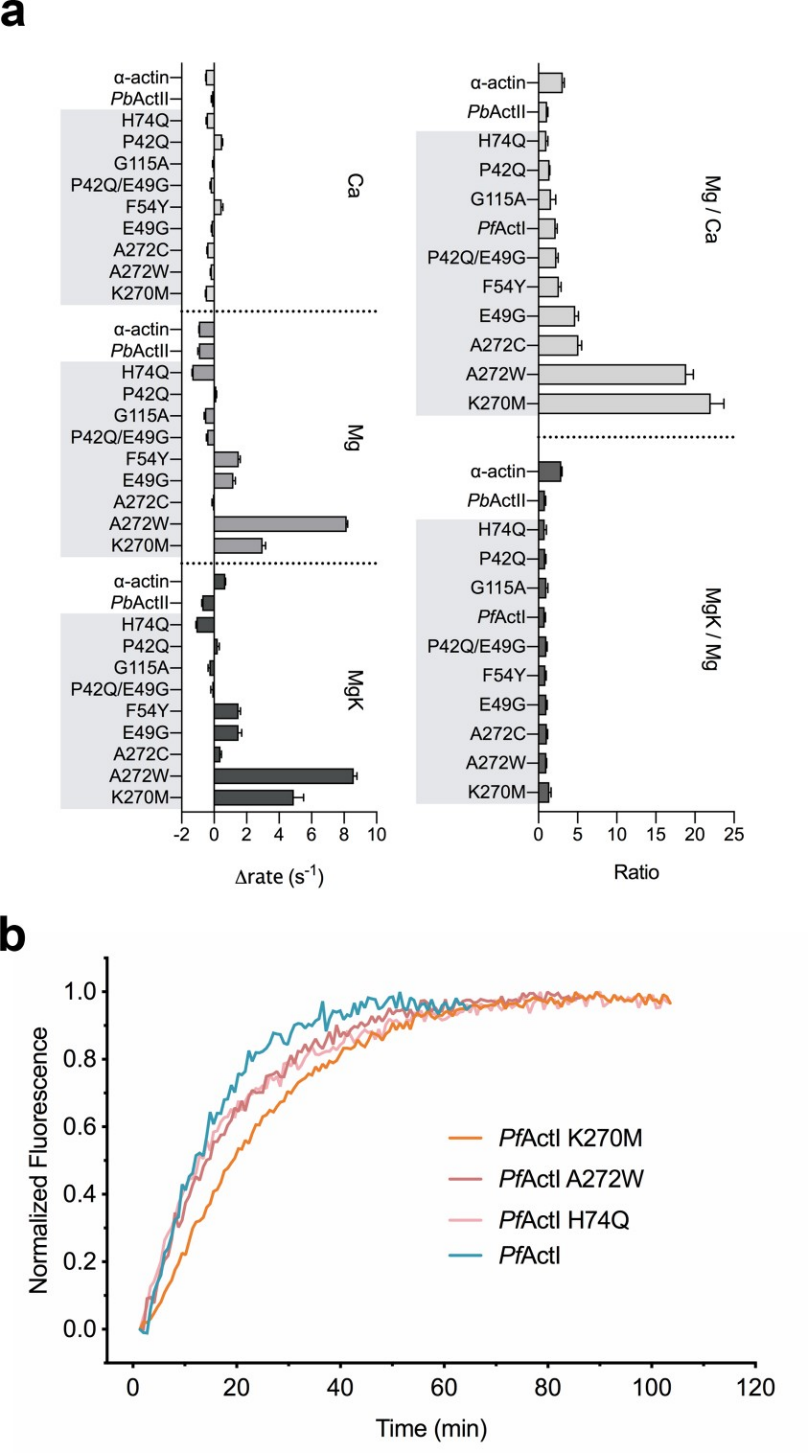
Phosphate release rates and elongation kinetics of *Plasmodium* and canonical actins. (A) Phosphate release rates of *Pf*ActI, *Pb*ActII, and α-actin as well as a collection of mutants of *Pf*ActI, expressed as rates compared with *Pf*ActI wild type by subtraction (left) and as ratios of Mg/Ca or MgK/Mg (right). In the text and S2 Table, the ratios are referred to as “activation.” The underlying data for this figure can be found in S1 Data. (B) Polymerization of 10% pyrene-labeled *Pf*ActI, *Pf*ActI K270M, *Pf*ActI A272W, and *Pf*ActI H74Q seeded with α-actin nuclei. *Pb*ActII, *Plasmodium berghei* actin II; *Pf*ActI, *P*. *falciparum* actin I.

In skeletal muscle α-actin, conformational changes upon polymerization activate nucleotide hydrolysis in the actin protomers, and the subsequent P_i_ release leads to destabilization of the “aged” filament [14,15]. Throughout this text, we refer to this activation of P_i_ release or nucleotide hydrolysis by the word “activation” unless otherwise stated. Actins are predominantly purified in their nonphysiological Ca^2+^ bound form, due to improved stability and higher critical concentration of polymerization. In physiological conditions, actin binds Mg^2+^ and is therefore mostly studied in this state. Polymerization is fastest and the critical concentration the lowest in physiological conditions with Mg^2+^ and K^+^ [7]. Critical concentrations of canonical actins and *Pf*ActI in such polymerizing conditions are similar [13]. In addition, P_i_ release rates have been studied earlier for canonical actins as well as *Pf*ActI and *Plasmodium berghei* actin II (*Pb*ActII). Filamentous α-actin releases P_i_ at rates of 14.8 × 10^−4^ s^−1^ during the elongation phase of polymerization and 0.15 to 0.47 × 10^−4^ s^−1^ once equilibrium (steady state) between polymerization and depolymerization has been reached [19,20] (S1 Table). By comparison, P_i_ release rates in equilibrium measured from *Pf*ActI and *Pb*ActII were >1.3 × 10^−4^ s^−1^ in the presence of Ca^2+^ and Mg^2+^ [9]. These measurements were conducted above the critical concentration of either filament end in the ATP state (1.5 μM for the barbed end, 4.5 μM for the pointed end in 1 mM Mg^2+^, as measured for α-actin [7]). To further characterize the relationship between phosphate release and polymerization, we measured P_i_ release rates from *Pf*ActI, *Pb*ActII, and α-actin in 0.2 mM Ca^2+^, 1 mM Mg^2+^, and 4 mM Mg^2+^/50 mM K^+^ at protein concentrations around 1 and 3 to 6 μM each. Contrary to α-actin, P_i_ release rates of the parasite actins did not increase in the polymerizing MgK conditions at low actin concentrations (Fig 1A, S2 Table). This was true also for higher concentrations of *Pf*ActI but not for *Pb*ActII (S3 Table). At higher concentrations, P_i_ release from *Pf*ActI was instantaneous with no identifiable lag phase in any of the conditions, whereas P_i_ release curves from *Pb*ActII and α-actin showed a lag phase in Mg and MgK states (S1 Fig). This is in line with our earlier report, in which spontaneous polymerization of *Pf*ActI showed a very short or nonexistent lag phase [13]. These data suggest that the coupling of nucleotide hydrolysis and P_i_ release with polymerization is different in *Pf*ActI compared with canonical actins and *Pb*ActII.

### Steps of ATP hydrolysis and phosphate release in *Pf*ActI can be followed in crystallo

**Fig 2:**
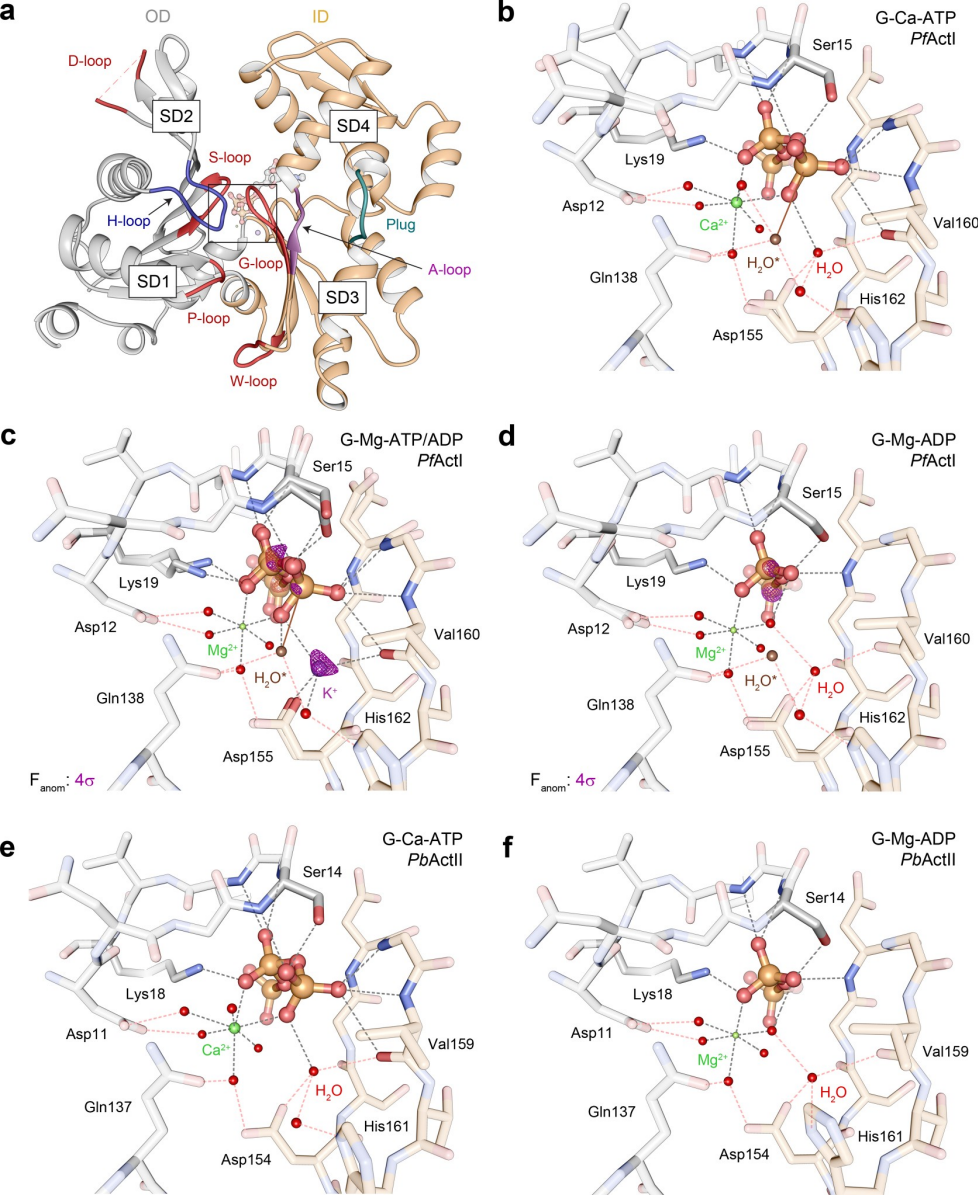
Active site configurations in the *Pf*ActI and *Pb*ActII structures. (A) Overview of the Mg-ATP/ADP-*Pf*ActI monomer with the D loop, S loop, H loop, G loop, P loop, and W loop as well as the plug and A loop indicated. The region of interest enlarged in the other panels is boxed. (B–D) *Pf*ActI structures in the (B) Ca-ATP [9], (C) Mg-ATP/ADP, and (D) Mg-ADP states. (E, F) *Pb*ActII structures in (E) Ca-ATP [9] and (F) Mg-ADP states. In all panels, hydrogen bonds with ATP, ADP, or ions are indicated with black dashed lines and the outer shell hydrogen bonding via water molecules with red dashed lines. In (B, C), the brown solid line indicates the nucleophilic attack vector of the putative catalytic water [21] (H_2_O*). In (C, D), anomalous difference density is shown as a purple mesh at a 4-σ contour level. The ID and OD are colored in orange and gray, respectively, in all panels. ID, inner domain; OD, outer domain; *Pb*ActII, *P*. *berghei* actin II; *Pf*ActI, *P*. *falciparum* actin I; SD, subdomain.

Since the major activation of P_i_ release from *Pf*ActI is caused by Mg^2+^, we decided to study the process in detail by analyzing crystal structures of monomeric *Pf*ActI and *Pb*ActII in the Mg state and compare those to the published high-resolution structures of the Ca states [9] (Fig 2, S4 Table). Both *Plasmodium* actins were crystallized as complexes with mouse gelsolin segment 1 (hereafter gelsolin) in the presence of 0.5 mM ethylene glycol-bis[2-aminoethyl ether]-N,N,N’,N’-tetraacetic acid (EGTA) and 1 mM MgCl_2_ as well as 200 mM potassium thiocyanate from the crystallization solution (S3 Table). In addition to the wild-type proteins, we also determined several structures of 4 *Pf*ActI mutants (A272W, H74Q, F54Y, and G115A) in different nucleotide states—altogether 10 structures—which will be discussed in the sections below.

The wild-type crystals diffracted to high resolution (1.2–1.85 Å, S5 and S6 Tables), enabling a detailed structural analysis. Actin-gelsolin complexes crystallized in the presence of Mg^2+^ may undergo complete or no hydrolysis of ATP [16,21,22]. To our surprise, in Mg-*Pf*ActI crystals from which diffraction data were collected soon after crystal formation, a model with partial occupancies for both ATP and ADP (and associated conformational changes in the protein such as Ser15) in the active site was found to best explain the data (Fig 2C, S2 Fig). We call this model Mg-ATP/ADP-*Pf*ActI. Only after aging the crystals for several months could we obtain data explained by an ADP-only model (Fig 2D), termed Mg-ADP-*Pf*ActI. Crystals grown after exchanging the nucleotide in solution after complex formation also contained a similar mixed nucleotide state. These findings were corroborated by anomalous difference density maps, in which peaks of >4 σ were found for Pα, Pβ, and Pγ in Mg-ATP/ADP-*Pf*ActI but only for Pα and Pβ in the Mg-ADP-*Pf*ActI. It therefore seems that gelsolin inhibits nucleotide exchange in *Pf*ActI as in α-actin [23], and that ATP hydrolysis happens in crystallo, with no exchange of the nucleotide with the solvent. The pH dependence of P_i_ release from free *Pf*ActI monomers was 60% to 114% at the crystallization pH range of 5.7 to 6.5 relative to the standard assay conditions at pH 7.5 (S3 Fig). Despite the mixed nucleotide state, we were unable to locate free P_i_ anywhere within the structure, even after soaking the Mg-ADP-*Pf*ActI crystals in P_i_. Contrary to *Pf*ActI, Mg-*Pb*ActII crystals contained only ADP just 2 weeks after crystallization, despite showing a slightly lower P_i_ release rate in solution than *Pf*ActI (S1 Fig and S3 Table). Thus, the effects of gelsolin and/or the crystalline environment apparently slow down the hydrolysis but not the P_i_ release rate of *Pf*ActI. This combination of high resolution and slow hydrolysis provides a convenient window to visualize the structural changes upon ATP hydrolysis, P_i_ release, and polymerization.

The overall structures of the different nucleotide states of *Pf*ActI appear very similar, but principal component analysis (PCA) with a set of 147 unique actin structures identified 2 conformational shifts during the reaction pathway (S4 Fig, S1 Movie): (i) opening of the nucleotide-binding cleft and (ii) slight flattening upon inclusion of Mg^2+^, followed by twisting of the monomer upon completion of hydrolysis. A data set comprising only *Plasmodium* actins shows a similar trend (S4 Fig), although principal component (PC)2 in this data set depicts a change in SD2 and not so much in SD1, as in the full data set (S1 Movie). The twist angles of the mass centers of the SDs (θ) were used as an independent measure and showed angles of 19.0°, 17.9°, and 20.0° for Ca-ATP, Mg-ATP/ADP, and Mg-ADP structures, respectively (S7 Table). The opening-closing motion was not evident from distances of the mass centers of SD2 and SD4 (d_2–4_) or phosphate clamp distances (b_2_) as defined before [24]. However, anisotropic B factors indicate a directional destabilization of SD2 toward SD4 (S5 Fig). It has to be kept in mind that all these crystal structures contain gelsolin bound to the cleft between SDs 1 and 3, which likely has an effect on both the twist and the opening motion of the actin monomer. We expect gelsolin to limit the flexibility of the domains with respect to each other, and thus the direction of these movements could be taken as indicative of the real situation, with the magnitude likely smaller than in a free actin monomer. A comparable data set of *Dictyostelium discoideum* actins is characterized in PCA by a combination of opening and twisting upon inclusion of Mg^2+^ and a reversal of the opening upon completion of hydrolysis [16].

### *Pf*ActI binds potassium during ATP hydrolysis

In the Mg-ATP/ADP-*Pf*ActI, we found excess electron density, not explained by water, between the side chain of Asp155, the backbone nitrogen of Gly157, and the backbone carbonyl of Val160 (Fig 2C and S2 Fig). An anomalous difference density map revealed a 6.5-σ peak at this site (Fig 2C). In other Mg-actin structures, no other metals have been identified in the active site besides the divalent cation. Considering ions present, coordination distances, and geometry as well as anomalous scattering lengths at the used wavelength (1.032 Å), K^+^ is the most likely explanation for this density. Furthermore, this site corresponds to one of the K^+^-binding sites identified in the homologous Heat shock cognate 71 kDa protein (Hsc70) nucleotide-binding domain [25]. We therefore modeled K^+^ at this site with a final refined occupancy of 0.7, which is close to the occupancy of ATP (0.8). Considering all the possible ions present in the crystals, the only other possibilities in addition to K^+^ would be Mg^2+^ or Cl^−^. We excluded Mg^2+^ based on coordination geometry. However, distinguishing between K^+^ and Cl^−^ is more difficult. To address this, we refined a chloride ion at this site and subsequently analyzed the models with K^+^ and with Cl^−^ using the CheckMyMetal server [26]. The results showed that K^+^ is coordinated in a tetrahedral configuration, whereas Cl^−^ is unliganded, which favors our assignment of K^+^. In addition, because of the coordinating negative side chain (Asp155) and the negatively charged phosphate tail of ATP, an anion would be very unlikely at this site.

The active site of actin is highly conserved, including the residues coordinating this K^+^. Yet there is no evidence of K^+^ or any other ions at this site in published actin structures, other than the Cd-ATP-*Pf*ActI structure [27], where Cd^2+^ was refined at this site. The Mg-ADP structure does not contain excess electron density or anomalous difference density at this site (Fig 2D), despite showing anomalous difference density for the Pα and Pβ atoms of ADP. This suggests that K^+^ leaves the active site upon P_i_ release. Because K^+^ does not activate P_i_ release from *Pf*ActI (Fig 1A, S2 and S3 Tables), this interaction most likely does not directly influence the mechanism of P_i_ release in *Pf*ActI but may rather be relevant for hydrolysis.

### Nonmethylated His74 and Lys270 play ping-pong on the A loop in *Pf*ActI

Three loops in the actin fold are considered primary sensors of the nucleotide state (Fig 2A): the S loop (residues 11–16 [28–30]), the H loop (residues 70–78 [29]), and the G loop (residues 154–161 [30]). Other, more distant sensors of the nucleotide state are the W loop (residues 165–172 [31]), the D loop (residues 38–52 [28–30]), and the C terminus (residues 349–375 [32]). The foremost nucleotide state sensor in canonical actins is Ser14 in the S loop, whose side chain rotates toward the β-phosphate of ADP upon P_i_ release. The conformation of the corresponding Ser15 in *Pf*ActI moves from the ATP state [9] through a double conformation with occupancies 0.8/0.2 in the ATP/ADP state to a complete ADP conformation (Fig 2B–2D). This conformational switch is further propagated to the flipping of the peptide bond between Glu73 and His74 in the nearby H loop (S6 Fig), as seen also in *Pb*ActII and the uncomplexed ATP and ADP structures of several actin structures [16,28,30].

**Fig 3:**
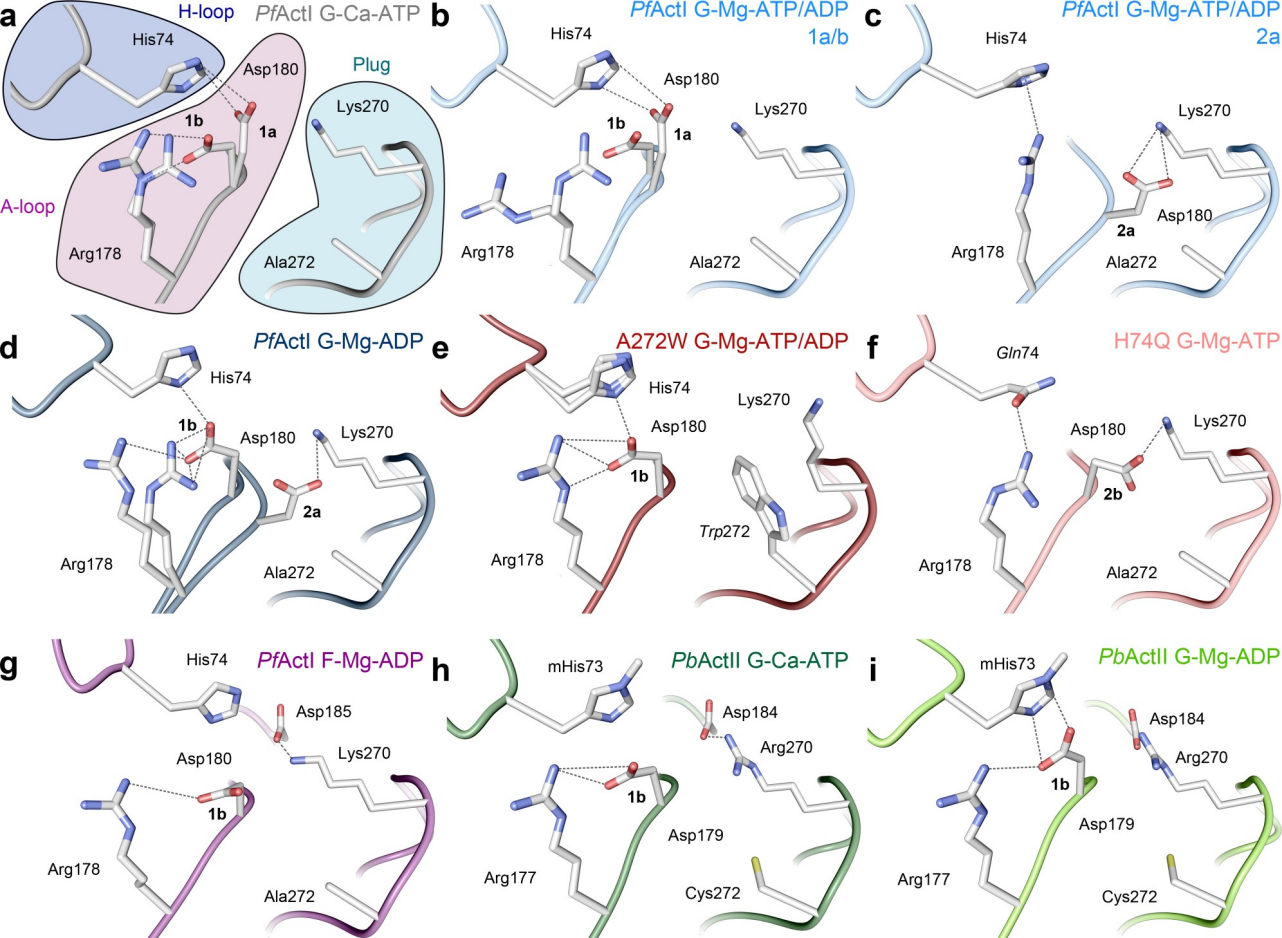
Orientation of the A loop in *Pf*ActI and *Pb*ActII. (A–D) Wild-type *Pf*ActI in the (A) Ca-ATP state [9] (1a and 1b), (B) Mg-ATP/ADP state (1a and 1b), (C) Mg-ATP/ADP state (2a), and (D) Mg-ADP state (1b and 2a). Note that panels (B) and (C) represent alternative conformations from the same crystal structure. (E–F) *Pf*ActI mutants (E) A272W in the Mg-ATP/ADP state (1b) and (F) H74Q in the Mg-ATP state (2b). (G) Wild-type *Pf*ActI in the F state [10] (1b), stabilized by jasplakinolide (not depicted). (H–I) Wild-type *Pb*ActII in the (H) Ca-ATP [9] (1b) and (I) Mg-ADP states (1b). In (H), His73 is methylated for consistency even though it is not in the deposited model. The most probable ionic and hydrogen bonding interactions are indicated with dashed lines. The conformers of the H loop are attributed to each conformation based on overlap of van der Waals radii as well as distances and geometry for hydrogen bonding. F, filamentous; G, globular; mHis73, methylated His73; *Pb*ActII, *Plasmodium berghei* actin II; *Pf*ActI, *P*. *falciparum* actin I.

Asp180 is located in a short loop following β14 (S7 Fig), sandwiched between the H loop and the plug residues, including Lys270 (Figs 1A and 3). We call this loop the A loop because of its anionic nature (described below), its central residues being arginine and aspartate, and because its relevance is here described from an apicomplexan parasite. The A loop serves as a linker between SD3 and SD4. In the Ca-ATP structure, the A loop resides close to the H loop (Fig 3A). Asp180 is in 2 conformations: either interacting with the Nδ of His74 (3.2 Å, conformation 1a) or oriented toward Arg178 (conformation 1b). In the Mg-ATP/ADP structure, the backbone of the A loop has a second conformation (conformation 2a) with an occupancy of 0.4 (Fig 3C). In the Mg-ADP structure, only conformations 1b and 2a are present at equal occupancies. B factors match the environment in both Mg structures (S8 Fig), and the occupancies are in agreement with the estimated protonation state (55%) of a histidine side chain in solution at pH 6.0. In conformation 2a, Asp180 forms a salt bridge with Lys270. In conformation 1a, Asp180 moves to form a salt bridge with His74. Thus, the A loop is engaged in a ping-pong movement between the 2 positive charges. Conformation 1b is analogous to the position of the side chain in the jasplakinolide-stabilized *Pf*ActI filament model (Fig 3G) and in many canonical actin filament models [10,33–35].

Most model actins, except for that of *Saccharomyces cerevisiae*, presumably have a methylated His74 (*Pf*ActI numbering) in the H loop [36], although this is not evident from the majority of structures in the Protein Data Bank (PDB). Histidine methylation is a rare post-translational modification that lowers the pK_a_ of the side chain by donating electrons to the π-system and thus stabilizing the positive charge. Our crystal structures are of sufficiently high resolution to verify the previous observations that in native or recombinant *Pf*ActI, His74 is not methylated [12,13]. Curiously, recombinant *Pb*ActII expressed and purified similarly is methylated at this position (S2 Fig). In actins with a methylated histidine at this site, Nδ is mostly protonated and free to interact with the carbonyl of Gly159 (*Pf*ActI numbering), which together with Val160 is involved in coordinating the active site K^+^ (Fig 2C). As protonated histidines act as cations in electrostatic interactions and as π-systems in cation-π interactions, protonation constitutes a credible interaction switch between His74^+^/Asp180^−^ and His74/Arg178^+^, particularly for a nonmethylated histidine. A methylated histidine in canonical actins and *Pb*ActII would favor interactions of the A loop with the H loop.

Arg178 in the A loop participates in connecting the inner domain (ID) and the outer domain (OD). In the Mg-ATP/ADP structure, Arg178 moves toward the carbonyl groups of His74 and Pro110 in conformation 1b, thus connecting the P loop in SD1 (residues 109–114) and H loop in SD2 (S9 Fig). Conversely in conformation 2a, Arg178 interacts with His74 via a cation-π interaction, which only maintains the contact between SD3 and SD2. Because the 2 conformations of the A loop backbone (1a/b and 2a) are evident in the presence of Mg^2+^ but not with Ca^2+^ and are still present in the Mg-ADP structure, the movement of the loop is either connected directly to Mg^2+^ binding or is an indirect result facilitated by Mg^2+^ binding and the resulting accelerated P_i_ release.

### Structural differences in the Ca and Mg states of *Pb*ActII

According to PCA, Mg-ADP *Pb*ActII is less open and more twisted than the Ca-ATP form, situating toward the twinfilin–C complex [37] and the cofilin-decorated filament structure [38]. Measurements of θ, d_2–4_, and b_2_ support these findings (S7 Table). However, the largest changes appear in SD2, which has high B factors and relatively weak electron density (S10 Fig). The active site configurations in the Ca states are similar between *Pf*ActI and *Pb*ActII (Fig 2B and 2E). However, in the presence of Mg^2+^, the His161 side chain adopts a different conformation in *Pb*ActII than that seen in any of the structures of *Pf*ActI and most other gelsolin-bound structures in the PDB (Fig 2F). The exception to this is the *D*. *discoideum* actin structure in the presence of Li-ATP (1NMD), in which a similar conformation was proposed to be more amenable to hydrolytic activity [21]. However, the side chain is rotated 180° about the Cβ-Cγ bond in 1NMD compared with *Pb*ActII and most other actin structures. The new conformation of His161 in *Pb*ActII changes the water network by occupying the space of one of the waters coordinating the active site K^+^ in *Pf*ActI. In canonical F actin, His161 adopts a conformation similar to that seen in *Pb*ActII but even closer to Pγ [18,35].

There is no evidence of conformations 1a or 2a in the *Pb*ActII Mg-ADP structure (Fig 3H and 3I). This can be rationalized as follows: (i) methylation of His73 ensures that it is mostly protonated and therefore repels Arg177, interacting with Asp179; (ii) Gly115 of *Pf*ActI is threonine in *Pb*ActII, and the G115A mutant also lacks conformation 2a (see below); and (iii) Ala272 of *Pf*ActI is cysteine in *Pb*ActII, which may sterically block the backbone position of conformation 2a. The fact that the alternative conformations of the A loop have not been built in the majority of actin structures does not unambiguously prove that they would not exist, and indeed in several cases, this loop has high B factors. However, based on available data, we expect that a stable conformation 2a may be unique to *Pf*ActI and that *Pb*ActII resembles canonical actins in this respect.

### Canonical-type K270M mutation in *Pf*ActI hyperactivates phosphate release and stabilizes filaments

**Fig 4:**
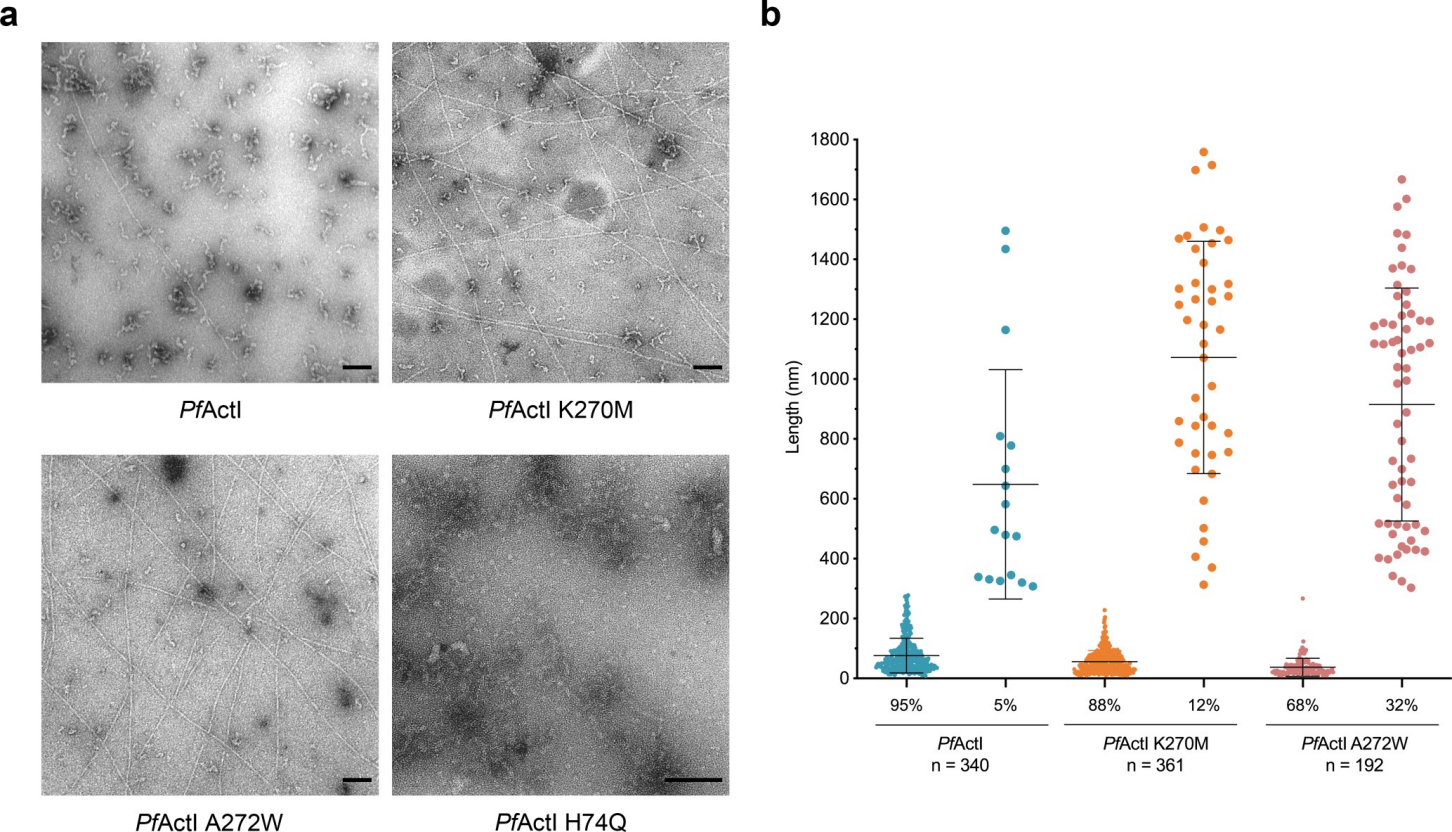
Abundance of long filaments is increased in *Pf*ActI mutations blocking conformation 2a. (A) Representative electron micrographs of negatively stained, polymerized wild-type *Pf*ActI and the K270M, A272W, and H74Q mutants. Wild-type *Pf*ActI formed mostly irregular short filament-like structures with the occasional appearance of long filaments. The K270M mutant formed longer helical filaments at an over double frequency, whereas the A272W mutant formed long filaments 6 times as often as the wild type. No long helical filaments were observed for the H74Q mutant. (B) Quantitative analysis of filament lengths of wild-type *Pf*ActI and the mutants K270M and A272W. Note that the maximum length is defined by the field of view, and in reality, the long filaments are much longer than what was measured. The underlying data for this figure can be found in S2 Data. Scale bars in (A) represent 100 nm. *Pf*ActI, *P*. *falciparum* actin I.

We proposed earlier that differences in the plug region and especially Lys270 (corresponding to Met269 in α-actin) are among the determining factors for *Pf*ActI filament instability [10]. Because Asp180 interacts with Lys270 directly, we generated a canonical-type K270M mutant. Indeed, this mutant formed many more long filaments in the absence of jasplakinolide than wild-type *Pf*ActI (Fig 4). Curiously, considering this stabilizing effect, the K270M mutation caused hyperactivation of the P_i_ release rate by Mg^2+^. This activation effect was manifested by a reduction of the rate in Ca conditions to α-actin levels and a moderate increase in Mg. Furthermore, in contrast to the wild type, K270M was no longer insensitive to K^+^ (S2 Table) and also showed a lag phase at high concentration (S3 Fig), thus behaving essentially as α-actin but with a faster rate in Mg and MgK conditions. In order to rule out that the differences in the P_i_ release rates would be caused by an altered elongation rate in the mutant, we performed seeded polymerization assays to compare the elongation rates. The elongation rates of K270M and wild-type *Pf*ActI are essentially identical (Fig 1B), meaning that the increased P_i_ release rate is not due to faster elongation. Because the K270M mutation should make conformation 2a less favorable by disrupting the interaction with Asp180, these results can be taken as indication that conformation 2a is counterproductive to P_i_ release.

### Mutations affecting the conformational space of the A loop affect phosphate release in *Pf*ActI

As the A loop moves into conformation 2a to interact with Lys270, it fills a space otherwise occupied by water molecules. On the opposite side, Ala272 points toward the A loop (Fig 3A–3G). This alanine is conserved in *Tg*Act and in nearly all alveolates but is replaced by serine in most model actins and by cysteine in *Pb*ActII or asparagine in *Arabidopsis thaliana* actin 1 (S7 Fig). We reasoned that if the disappearance of the positive charge by the K270M mutation changed the P_i_ release dramatically, P_i_ release might be directly related to the conformation of the A loop. Thus, large side chains at position 272 that affect the movement of the A loop should also modulate the P_i_ release rate. We therefore prepared A272C and A272W mutants—the first to provide a side chain of moderate size, also mimicking *Pb*ActII, and the second to block the movement of the loop completely, both presumably favoring conformation 1a/b. The A272C mutant caused a moderate 5.1-fold activation upon Mg^2+^ binding, whereas the A272W mutant showed a large 18.9-fold activation and the largest observed rate (9.78 ± 0.06 × 10^−4^ s^−1^) in Mg conditions (Fig 1A, S2 Table). As with K270M, the increased P_i_ release rate in the A272W mutant is not due to faster elongation (Fig 1B), and there are more long filaments of this mutant at equilibrium (Fig 4).

The A272W structure in MgK conditions resembles overall the mixed structure (root-mean-square deviation (RMSD)(Cα) = 0.269) more than the Mg-ADP structure (RMSD(Cα) = 0.410) and is positioned close to the Ca-ATP structure in the PCA analysis. The A loop is forced into conformation 1b by the Trp272 side chain (Fig 3E). Glu73 is in a double conformation, one similar to the Mg-ADP structure and another to that of the Mg-ATP/ADP structure (Fig 3E, S6 Fig). In addition to limiting the conformational space of the A loop, Trp272 forces Lys270 away from the Asp180 side chain and toward the solvent, widening the gap between His74 and Lys270 from 7.7 to 10.4 Å and only slightly altering the conformation of residues Leu268-Asn281 (RMSD = 0.27 Å, Mg-ATP/ADP-*Pf*ActI compared with Mg-ATP/ADP-*Pf*ActI-A272W; Fig 3E). The occupancy of ATP in the active site of this relatively fresh crystal is only 0.3 (S5 Table).

To generate a mutant that would favor conformation 2a of the A loop, we further prepared a neutralizing H74Q mutant, which negates the charge on the histidine side chain, forcing an unfavorable interaction of the glutamine with Asp180. This mutant was severely compromised in terms of P_i_ release, with α-actin levels of P_i_ release in the Ca state (0.27 ± 0.03 × 10^−4^ s^−1^) and no activation by either Mg^2+^ or K^+^ or by using a higher protein concentration (Fig 1A, S2 Table). Moreover, H74Q did not form any long filaments, even though its elongation rate appeared rather similar to the wild type. (Figs 1B and 4). In this mutant (MgK conditions), the Asp180 side chain is oriented away from Gln74, which interacts with Arg178 (Fig 3F). However, the backbone of the loop did not adopt conformation 2a, and we therefore call this conformation 2b, because the carboxylic acid group of the Asp180 side chain occupies the same space as that in conformation 2a, preserving the interaction with Lys270 (Fig 3F).

### Arg184 interactions with the H loop in SD2

**Fig 5:**
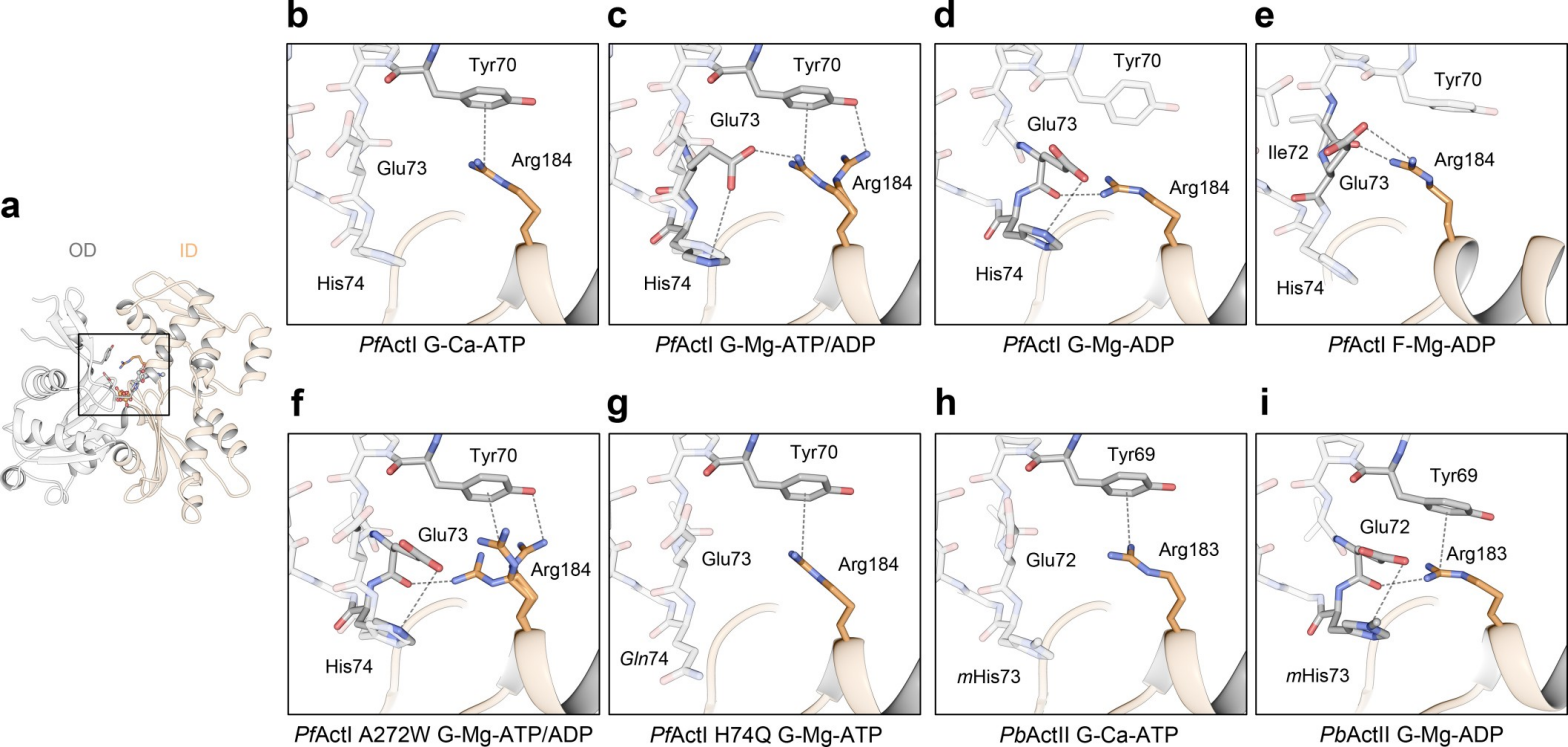
Conformation of the H loop residues 70–74 as well as the domain cleft spanning Arg184 in *Pf*ActI and corresponding residues 69–73 and Arg183 in *Pb*ActII. (A) Overview of the wild-type *Pf*ActI monomer in the Ca-ATP state [9] for positional reference. (B–E) Wild-type *Pf*ActI in the (B) Ca-ATP state [9], (C) Mg-ATP/ADP state, (D) Mg-ADP state, and (E) F state [10]. (F–G) *Pf*ActI mutants (F) A272W in the Mg-ATP/ADP and (G) H74Q in Mg-ATP states. (H–I) Wild-type *Pb*ActII in the (H) Ca-ATP [9] and (I) Mg-ADP states. The ID and OD are colored in orange and gray, respectively, in all panels. His73 of *Pb*ActII in (H) is methylated for consistency even though it is nonmethylated in the original PDB entry. Interatomic distances amenable to ionic interactions or hydrogen bonding (≤4 Å) are shown as dashed lines. F, filamentous; G, globular; ID, inner domain; mHis73, methylated His73; OD, outer domain; *Pb*ActII, *Plasmodium berghei* actin II; *Pf*ActI, *P*. *falciparum* actin I.

Interactions across the interdomain cleft mediate twist angle stability and the openness of actin [39]. Upon ATP hydrolysis in *Pf*ActI, Glu73 in the H loop undergoes a conformational shift, whereby the backbone is flipped and the side chain orients toward the ID and interacts with Arg184 (Fig 5A–5E, S9 Fig). This conformational shift happens also in *Pb*ActII (Fig 5H and 5I) and in several canonical actin structures [16,28,30]. In Ca-ATP-*Pf*ActI, Arg184 is engaged in a cation-π interaction with Tyr70. This interaction is preserved in the mixed structure but is dissipated in the pure Mg-ADP state (Fig 5B–5D), after an interaction transfer of Arg184 from Tyr70 to the flipped backbone carbonyl of Glu73. In F-*Pf*ActI, the interaction between Arg184 and Glu73 is enhanced by a hydrogen bond between Arg184 and the Ile72 carbonyl. In the *Pf*ActI H74Q and A272W mutants, the conformations in this area resemble those of the Ca-ATP (in H74Q) and Mg-ADP (in A272W) states (Fig 5F and 5G, S6 Fig).

### The effects of canonical-type mutations in the D loop on phosphate release

The major substitutions in the D loop of *Pf*ActI are Pro42, Glu49, and Phe54 (Gln41, Gly48, and Tyr53 in α-actin; S7 Fig). Tyr53 is a conserved phosphoregulation site in canonical actins [22], whereas the other 2 sites are interesting because of their possible conformational effects. These residues are invisible or only barely visible (in the case of Phe54) in the crystal structures. However, in the filament, the tip of the D loop of *Pf*ActI differs from canonical actins [10]. We therefore measured P_i_ release rates for the mutants F54Y [9], P42Q, E49G, and the double mutant P42Q/E49G of *Pf*ActI. P42Q and E49G showed opposite effects in Mg^2+^ activation with P42Q reducing and E49G increasing it, but both were similarly insensitive to K^+^ (Fig 1A, S2 Table). However, the negative effect of P42Q is due to an increase in the Ca rate compared with wild type, whereas the positive effect of E49G on Mg^2+^ activation is caused by both reduced rate in Ca and an increased rate in Mg. The double mutant has reduced Mg^2+^ activation with levels indistinguishable from the wild type while still remaining insensitive to K^+^. Thus, it seems to be dominated by the effect of E49G in the Ca state and shows a compounded negative effect that is not shown by either of the mutations alone.

At high concentration (10 μM), the F54Y mutation reduces the rate of hydrolysis in the Ca state to α-actin levels [9]. Here, we measured the rates at a concentration of 1 μM. The Mg^2+^- and K^+^-activation levels of F54Y were similar to the wild type, but the absolute rates were approximately doubled (Fig 1A, S2 Table). In the Ca condition, the F54Y mutant behaves similarly to P42Q (S3 Table), whereas the rates in the Mg and MgK conditions were most similar to the E49G mutant. Thus, these canonical-type mutations in the D loop area all have similar effects on P_i_ release. However, whereas P42Q and E49G directly affect the tip of the D loop in the filament, F54Y presents no foreseeable structural changes besides the added H bonds to Lys62 of monomer n and to Tyr170 of monomer n − 2 in the filament.

### G115A mutation structures the C terminus of *Pf*ActI

**Fig 6:**
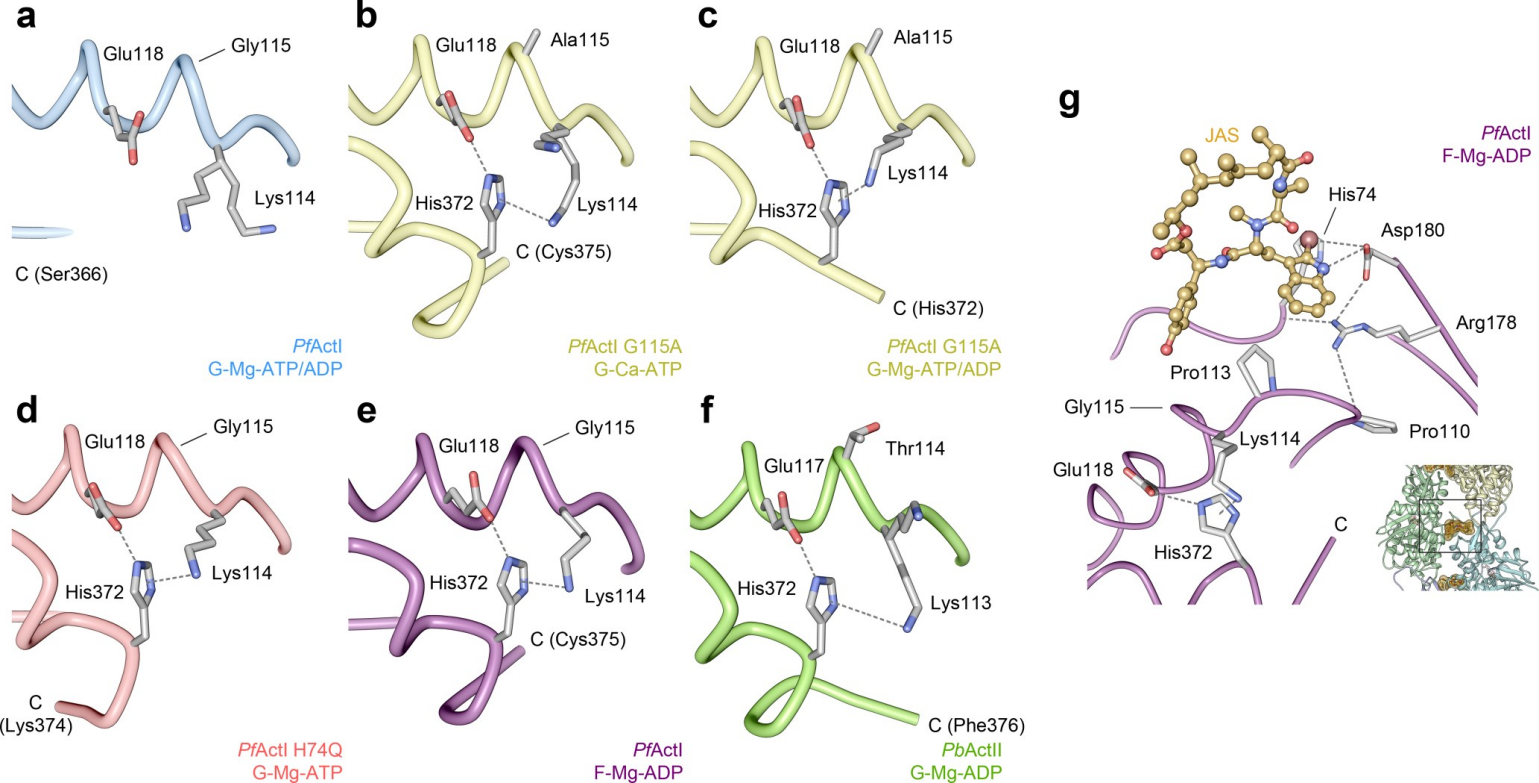
Interaction of the C termini of *Pf*ActI and *Pb*ActII with Lys114 (Lys113 in *Pb*ActII) and Glu118 (Glu117 in *Pb*ActII) of α3. (A) Wild-type *Pf*ActI in the Mg-ATP/ADP state has a disordered C terminus. The *Pf*ActI G115A mutant, in contrast, shows an ordered C terminus in the Ca-ATP state (B) and in the Mg-ATP/ADP state (C), similarly to the H74Q mutant in the Mg-ATP state (D). (E) Wild-type *Pf*ActI in the JAS-stabilized F state [10] and (F) *Pb*ActII in the Mg-ADP state also have stabilized C termini. The C-terminal His372 interacts with Lys114 and Glu118 of α3 due to the displacement of the N-terminal tip of the helix. In G115A, this is caused by the altered backbone conformation. In H74Q, the effect is likely indirect. In wild-type *Pf*ActI, the C terminus is not stabilized in any gelsolin-bound structure by the His372 interactions, which however are retained in the JAS-stabilized filament structure due to interactions of the P loop with the bromoindole moiety in JAS. In *Pb*ActII (F), residue 114 (corresponding to Gly115 in *Pf*ActI) is threonine and elicits a stabilization of the C terminus. (G) JAS interactions with the P loop and the A loop in the F-*Pf*ActI structure [10]. Interaction distances amenable to ionic or hydrogen bonding interactions (≤4 Å) are indicated with dashed lines. The inset in (G) shows the position in the filament. C, C terminus (with the terminal residue indicated); F, filamentous; G, globular; JAS, jasplakinolide; *Pb*ActII, *Plasmodium berghei* actin II; *Pf*ActI, *P*. *falciparum* actin I.

Gly115 in *Pf*ActI is located in the P loop of SD1 and is Thr, Ser, or Ala in other reference actins (S7 Fig). Nearby, Pro110 interacts with Arg178 in conformation 1b, and the backbone flexibility conveyed by Gly115 could control the positioning of this interaction. We previously generated a mutant G115A that did not rescue long filament formation in the absence of jasplakinolide but showed slightly longer filaments than the wild type in its presence [9]. We crystallized the mutant using the same conditions as the wild-type *Pf*ActI with either Ca^2+^ or Mg^2+^ to compare these structures. Unlike the wild type (Fig 6A), the C terminus of G115A is more ordered, with interpretable electron density up to Cys375 in the Ca^2+^ and up to His372 in the Mg^2+^ structure (Fig 6B and 6C). In contrast, all other structures of *Pf*ActI, with the exception of H74Q (Fig 6D) and the *Pb*ActI-α-actin D-loop chimera [9], have a disordered C terminus after Ser366.

The G115A mutation straightens α3 and moves the P loop slightly away from the C terminus. This in turn favors a cation-π interaction between Lys114 and His372 (3.7 Å) and a hydrogen bond between Glu118 and His372 (2.8 Å). In the wild type, the position of Lys114 does not allow both interactions to take place simultaneously, which is the likely reason for the disordered C terminus (Fig 6A). In filaments, this interaction is preserved with corresponding distances of 3.1 Å (Lys114-His372) and 3.0 Å (Glu118-His372; Fig 6E). *Pb*ActII, which has an ordered C terminus in both Ca and Mg states (Fig 6F), has a threonine in the corresponding position 114. The distances from Lys113 and Glu117 to His372 are 2.7 Å and 5.0 Å in Mg-ADP-*Pb*ActII. The altered position of the P loop does not extend to Pro110 and therefore does not directly influence the interactions of Arg178 at the interface of SD1 and SD2. Trp357 and Phe353 are in a double conformation in both structures, the former facilitating a recently identified cation binding site [40]. The conformations 1a and 1b of the A loop are evident in these structures, but conformation 2a is not visible in the Mg^2+^ structure. G115A has only slightly decreased P_i_ release rates in Mg and MgK conditions (Fig 1A, S2 Table).

## Discussion

### On the roles of ions in actin polymerization

The fortunate coincidence that our crystallization condition for both *Plasmodium* actins and the mutant forms contained K^+^ provided direct evidence of Mg^2+^-dependent K^+^ binding in the active site of *Pf*ActI. This is, to the best of our knowledge, the first experimental evidence of K^+^ in the active site of actin. The presence of K^+^ is in conjunction with the Mg-ATP state but not with the Ca-ATP or Mg-ADP states. Thus, K^+^ seems to be involved in hydrolysis and leave the active site together with the P_i_. Compared with the nonphysiological, inactive calcium-bound state, Mg^2+^ binding in the presence of K^+^ causes a slight flattening and possibly a tendency toward opening of the *Pf*ActI monomer, followed by a closing and twisting back upon hydrolysis. The slightly flattened conformation may well be the explanation why Mg-K-ATP actin is the fastest polymerizing actin species [7]. Conversely, Mg-K-ADP actin polymerizes weakly in canonical systems [7], and the twisting (i.e., moving further away from the F conformation) upon ATP hydrolysis, as seen for *Pf*ActI, may explain this. However, because the path of the G-F transition may have major intermediates that are off the linear path and cannot be captured by crystallographic analysis, the validity of the connection between polymerization propensity and twist of a G-actin structure remains to be confirmed. It should also be noted that the response of *Pf*ActI to ADP differs from canonical actins [9], and we do not yet completely understand the dependency of ATP hydrolysis and polymerization in the parasite actins.

A structural homolog of actin, Hsc70, has a conserved K^+^ binding site at the same location as *Pf*ActI [25]. The activity of Hsc70 decreases slightly in the presence of ammonium [41], which is in line with our previous finding that CH_3_COONH_4_ is able to “protect” *Pf*ActI from oligomerization, which in turn is dependent on ATP hydrolysis [9,13]. However, because *Pf*ActI did not respond to K^+^ in P_i_ release assays, the exact role of the active site K^+^ in P_i_ release remains to be investigated. The positive charge on the K^+^ may play a role in orientation of the γ-phosphate or the catalytic water or charge complementation of its conjugate base OH^−^ in the reaction pathway, as has been suggested for Hsc70 [42]. Unlike Hsc70, however, the presence of K^+^ is not mandatory for hydrolysis in *Pf*ActI. Yet its presence may challenge previous hydrolysis mechanisms proposed based on simulations [43,44].

### The A loop enables fragmentation but at the same time likely also contributes to increased nucleation

Apicomplexan microfilaments are short but display a relatively normal critical concentration of polymerization, which means that the filament length distribution must result from the overabundance of nucleation, fragmentation, or both. Because the lag phase is very short [13], increased nucleation likely contributes. However, we believe fragmentation is at least equally important, and these 2 are likely interconnected. The conformation of the A loop clearly connects to the stability of long filaments in *Pf*ActI. This is evidenced by increased proportion of long filaments when conformation 1a/b is favored by mutations K270M and A272W and, on the other hand, by a complete lack of long filaments in the 2a-favoring H74Q mutant. The K270M mutant also releases phosphate quantitatively faster and qualitatively similarly to α-actin. In the *Pf*ActI filament model, conformation 2a is not seen, likely because jasplakinolide binds both Arg178 and Asp180, fixing them in a stable conformation. In its absence, the filament structure would permit this conformation. Because these mutations affecting phosphate release rate and filament length do not significantly modulate polymerization kinetics, it is likely that a mechanism distinct from simple weakening of interprotomer contacts exists. However, because short filaments are still present in K270M and A272W, other factors such as the ones at the base of the D loop (discussed below) may be involved. In vivo, the mutation K270M is lethal in the blood stages of the *Plasmodium* life cycle, which serves to illustrate the significance of filament length to parasite survival [45].

The interplay between the H loop, the A loop, and the plug is complex, but our data provide important insights into how the movement of this triad connects to the mechanism of P_i_ release and (de)polymerization. P_i_ release is strongly influenced by the conformational distribution of the A loop into the 2 configurations 1a/b and 2a/b, as we show by P_i_ release measurements and structures. Conformation 2b is counterproductive to P_i_ release, whereas elimination of 2a by steric hindrance (as in the mutants A272W and A272C) or by charge neutralization (K270M) favors P_i_ release, suggesting that interactions of the A loop with the H loop and the P loop are required for native activity levels. Methylation of His73/74 and the resulting change in side-chain charge distribution is a key modulator of P_i_ release. A methylated histidine, as found in most actins, is approximately 11-fold more protonated in the cellular pH than a nonmethylated histidine would be. The only other species with a nonmethylated histidine at this position, and for which there are structures available, is *S*. *cerevisiae*, which—like *Pf*ActI—has a shorter lag phase of polymerization and no lag in phosphate release upon polymerization [46]. However, in structures of *S*. *cerevisiae* actin (*Sc*Act), conformation 2a/b is not present, possibly due to the presence of Leu269 and Ala114 instead of Lys270 and Gly115 [47].

F-actin–like interactions in the Mg-ATP state can be considered favorable for polymerization. We consider interactions spanning the cleft between ID and OD on the back face of the monomer the most favorable for flattening and thus nucleation and polymerization. There are only 2 such interaction sites: (i) between Arg184 of SD4 and Tyr70 and Glu73 of SD2 (Fig 5) and (ii) between Arg178 of SD3 and Pro110 and His74 of SD1 and SD2, respectively (S9 Fig). In (i), the interaction of Arg184 via a cation-π interaction to Tyr70 is supplemented by an ionic bond with Glu73 in the Mg-ATP/ADP structure, followed by a movement of Glu73 toward SD2 and a consequent hydrogen bond to the backbone of Ile72 in the F state. Yet, although the polymerization rate of the β-actin R183W mutant was significantly decreased [39], the α-actin R183G mutant displayed unaltered polymerization kinetics [48]. In (ii), the Arg178 interaction is absent in Ca-ATP actin but present in conformation 1b of Mg-ATP/ADP-*Pf*ActI. The interaction is preserved between His74 and Arg178 and further strengthened by hydrogen bonding to the carbonyl of Leu111. An R177H mutant in yeast actin results in an extended lag phase in polymerization [49], which corroborates that this interaction promotes nucleation. Arg177 is also the site for polymerization-inhibiting ADP ribosylation by iota toxins [50,51].

**Fig 7:**
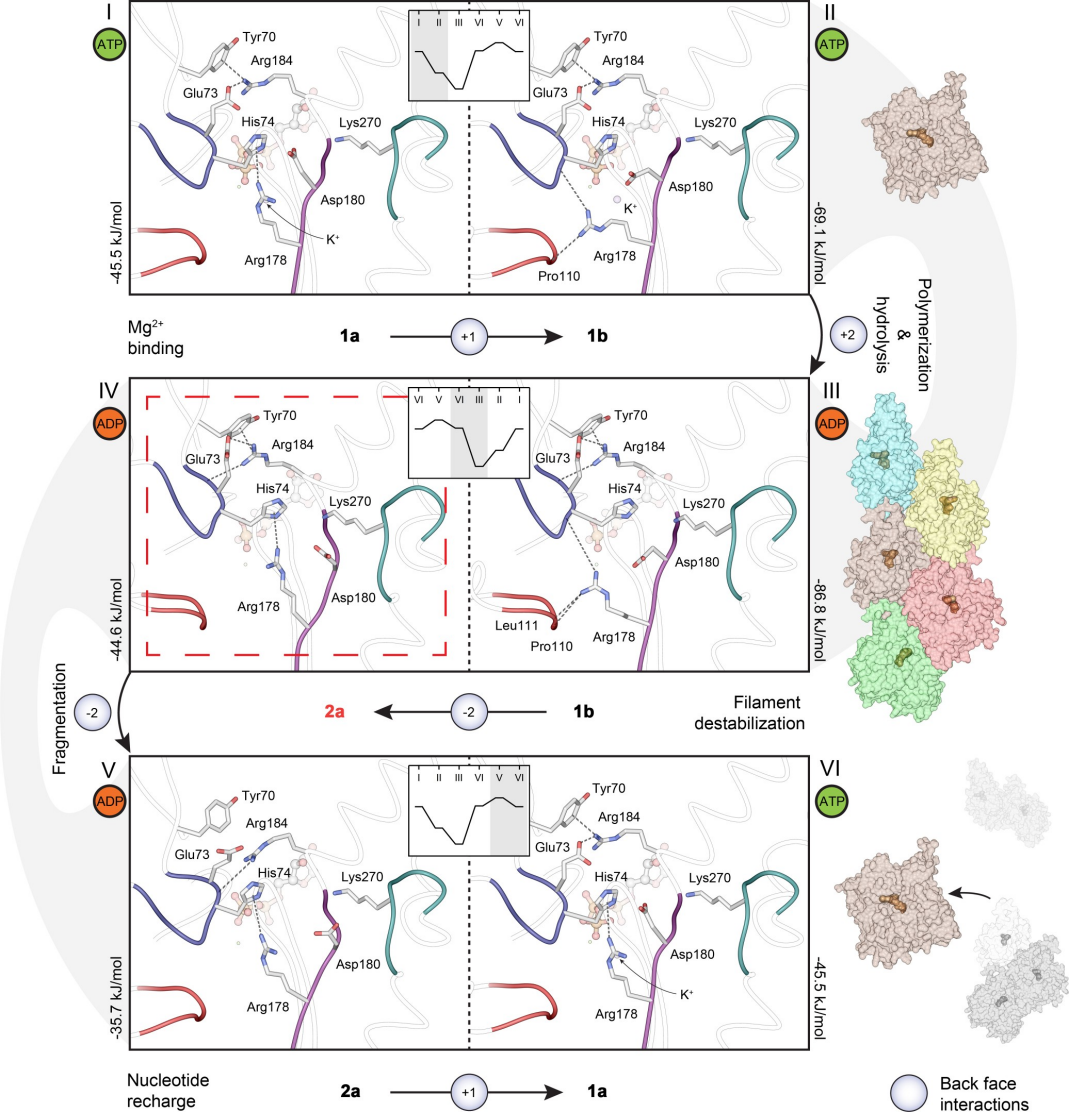
Mechanistic model of conformational changes in the *Pf*ActI monomer during polymerization, fragmentation, and nucleotide recharge. The exchange of Ca^2+^ to Mg^2+^ in vitro causes a conformational change from conformation 1a (I) to 1b (II), conferring 2 new back-face interactions that stabilize an F-like conformation. Upon polymerization (III), 2 new interactions are formed (Arg178 to backbone of Leu111 and Arg184 to backbone of Glu73), further stabilizing the flat conformation. In F-*Pf*ActI, ATP is hydrolyzed to ADP, and the P_i_ is released without major rearrangements [18], causing a further reduction in interactions spanning the ID–OD cleft via the G and S loops (loss of 5 hydrogen bonds between *Pf*ActI and Pγ; not depicted). In a hypothetical model of F-*Pf*ActI, in which conformation 2a is adopted (IV), 2 interactions formed by the adoption of 1b (II) are broken, causing a destabilization of the OD in respect to the ID, promoting a filament break. Upon fragmentation and dissociation of the monomer from the newly formed pointed end, conformation 2a is retained (V) in the ADP-*Pf*ActI monomer, the nucleotide is exchanged, and conformation 1a is reassumed (VI). Changes in the number of interactions on the back face of the monomer (on the inside of the filament) across the ID–OD cleft are highlighted in blue circles. Total interactions (hydrogen bonds, ionic interactions, and cation-π interactions) across the ID–OD cleft are 1, 3, 5, 2, and 1 in G-Mg-ATP 1a, G-Mg-ATP 1b, F-Mg-ADP 1b, F-Mg-ADP 2a, and G-Mg-ADP 2a, respectively, excluding changes caused by loss of Pγ. In the insets, interaction energies from Arg178, Asp180, and Arg184 to H and P loops are indicated in graphical form, whereas the absolute interaction energies are indicated next to each panel. Note that the inset is mirrored in panels III and IV to reflect the overall flow of the figure. The underlying data for this figure can be found in S3 Data. F, filamentous; G, globular; ID, inner domain; OD, outer domain; *Pf*ActI, *P*. *falciparum* actin I; P_i_, inorganic phosphate.

Based on our observations, we propose a model for *Pf*ActI filament fragmentation (Fig 7, S2 Movie). In this model, conformation 2a in the naked *Pf*ActI filament severs the contact between the ID and OD, leading to destabilization of the monomer twist and filament contacts, eventually causing a break in the filament. The model provides an alternative, perhaps complementary, explanation to the electrostatic effects we presented based on the filament model [10] and would also explain the increased pelleting of native *Pf*ActI at low pH [8].

### On the role of the D loop

The 2 substitutions in the D loop (Pro42 and Glu49 in *Pf*ActI) contribute to the unstable nature of *Pf*ActI filaments. These mutations favor the unstable closed D-loop conformation [35] to such an extent that even in the presence of jasplakinolide, which forces the stable open D-loop conformation in α-actin, the *Pf*ActI filament adopts the closed conformation [10]. Pro42 and Glu49 are in close proximity to the stiffness and polymerization cation sites [52], which in turn are close to 2 substitutions in *Pf*ActI, namely, Gly200 and Phe54. Together, these residues seem to participate in a complex interplay that is likely one of the major components of filament instability in *Pf*ActI. Because P_i_ release of E49G is activated 2.2-fold more by Mg^2+^ than wild type, whereas the activation of P42Q/E49G and P42Q is equal or less, respectively, one can conclude that these mutations are complementary to each other and that conformational restrictions of the D loop and P_i_ release rates are reciprocally connected. Like K270M, the mutation P42Q is lethal in vivo, highlighting the fact that the conformational rigidity of the D loop is critical to parasite biology [45]. Additionally, the effects of the F54Y mutation on overall rates (but not the activation) show that this mutation has a role beyond post-translational modifications. Interestingly, structural information on P_i_ release seems to be “erased” from α-actin filaments by jasplakinolide, which is attributed to the D-loop conformation [35]. The fact that *Pf*ActI filaments can be stabilized by adding jasplakinolide into filaments after polymerizing to equilibrium [10] shows that the binding of jasplakinolide can overcome the effects of the constantly closed conformation of the D loop.

### Concluding remarks

Although there are several structural factors contributing to the unique properties of *Plasmodium* microfilaments, the A loop seems to be a major switch between stable and unstable filament conformations. As such, it would be responsible for faster breakdown of the filaments but, on the other hand, also for lowering the energy barrier for polymerization, leading to increased nucleation. There are no known actin-binding proteins that can directly affect this region of the filament, suggesting that this mechanism could be a major intrinsic determinant of filament lengths in vivo. Importantly, although less favorable due to increased protonation of the methylated His73, the lack of attraction between Asp179 and Met269, and the apparent absence of conformation 2a caused by the G115A substitution, the proposed mechanism could work also in canonical actins. As crystal structures represent low-energy states, it is possible that fragmentation in canonical actins proceeds through the same mechanism, simply less frequently.

## Materials and methods

Unless otherwise stated, all materials were purchased from Sigma (St. Louis, MO).

### Mutagenesis

*Pf*ActI mutants were generated by site-directed mutagenesis as described for F54Y and G115A by Vahokoski and colleagues [9]. Mutants A272W, A272C, H74Q, P42Q, E49G, and P42Q/E49G were prepared using similar methods as before, with different primers. All mutants were confirmed by capillary sequencing at the Biocenter Oulu Sequencing Core or at the Center for Medical Genetics and Molecular Medicine, Haukeland University Hospital, Bergen.

### Protein expression and purification

Wild-type and mutant *Plasmodium* actins were purified as described by Vahokoski and colleagues [9,13]. Briefly, insect-cell–expressed His-tagged *Plasmodium* actins were purified using Ni-NTA (Qiagen, Hilden, Germany) affinity chromatography, cleaved with a recombinantly expressed protease (tobacco etch virus [TEV] protease for *Pf*ActI and rhinoviral 3C protease for *Pb*ActII). The cleaved protein was then passed through a second Ni-NTA column to remove the His tag and uncleaved protein. Finally, the purification was finalized by gel filtration over a Superdex 200 column (GE Healthcare, Chicago, IL). Mouse gelsolin segment 1 was purified as described by Bhargav and colleagues [53] and included in actin samples (where applicable) before gel filtration at a 1.2-fold molar excess.

### Phosphate release assays

P_i_ release was measured using the 7-diethylamino-3-[N-(2-maleimidoethyl)carbamoyl]coumarin-labeled phosphate-binding protein (MDCC-PBP) biosensor [54,55] that produces a fluorescence signal upon P_i_ binding. To reduce P_i_, ATP, and ADP background, monomeric actins used for P_i_ release assays were pretreated with DOWEX 1X8 resin equilibrated with G_0_ buffer (10 mM HEPES [pH 7.5], 0.2 mM CaCl_2_, 0.5 mM TCEP) for 3 min at 298 K and further diluted using G_0_ buffer to 1.6-fold higher concentration than that used for measurements. Before initiating the kinetic measurements, components for the different conditions were supplied as 8-fold concentrated stocks such that the desired final concentrations of all components were reached. Final compositions of the 3 conditions were 10 mM HEPES (pH 7.5), 0.2 mM CaCl_2_, 0.5 mM TCEP, 50 μM ATP (Ca condition), Ca condition with added 1 mM MgCl_2_ (Mg condition), and Ca condition with added 4 mM MgCl_2_, 1 mM EGTA, and 50 mM KCl (MgK condition). Fluorescence was recorded using a Tecan Infinite M1000 plate reader (Tecan, Männedorf, Switzerland) and black 384-well plates (Greiner, Kremsmünster, Austria) at λ_ex_ = 430 nm and λ_em_ = 465 nm using bandpass filters with 5-nm bandwidth and a 10-s measurement interval. Fluorescence versus time data were converted to μM P_i_ by linear interpolation of a standard series of P_i_ and analyzed by linear regression at the linear portion of the kinetic curve. The slope of the regression line was then divided by the protein concentration measured after DOWEX treatment to yield the final rates. For the P_i_ release curves, the abscissa units are values of μM phosphate released divided by μM actin, which describes the average turnover of the hydrolytic cycle in each sample.

In the presence of Mg and in MgK, α-actin displays an initial lag phase, followed by an exponential P_i_ release curve, which, at high concentrations, plateaus close to the upper limit of the linear range of the system (S1 Fig). We therefore decided to consider only the first 2 phases for our analyses. We further calculated the activation of P_i_ release by Mg^2+^ and by K^+^ by dividing the Mg rate by the Ca rate in the former and the MgK rate by the Mg rate in the latter. These ratios are a sensitive measure for comparing actins to one another, because they are insensitive to changes in residual nucleotide contamination in the samples. These contaminants are of the order of <10% of the 50-μM ATP added to each reaction. Because the total nucleotide concentration is in a >50-fold excess over the nM-range dissociation constant of ATP to actin [56], we assume that in the assay, actin is saturated and not affected by small fluctuations in the nucleotide concentration. All plotting and analysis were performed in Prism 8.0.0 (GraphPad, San Diego, CA).

### Actin polymerization assays

*Pf*ActI wild type and mutants were labeled with N-(1-pyrene)iodoacetamide (Life technologies, Invitrogen, Eugene, OR) as described by Kumpula and colleagues [13]. Labeled and unlabeled actin samples were mixed in a volume ratio of 1:2 (labeled:unlabeled) by volume at a concentration of 8.1-μM actin to reach a labeling ratio of approximately 10%. Polymerization assays nucleated by α-actin filaments were prepared as described by Kumpula and colleagues and Pollard [13,57]. Briefly, 50 μl of the above mixture was transferred into each well followed by the addition of 100 μl of 0.75-μM polymerized muscle α-actin seed solution that was freshly prepared in 1.5× F buffer from a 5-μM stock to initiate the polymerization reaction. The resulting 150-μl reaction mixture contained 1× F buffer with final *Plasmodium* actin and α-actin concentrations of 2.7 μM and 0.5 μM, respectively. Measurements were carried out in a Tecan Spark 20M multimode microplate reader using black 96-well plates (Greiner), λ_ex_ = 365 nm (9 nm bandpass) and λ_em_ = 407 nm (20 nm bandpass), 5 flashes per measurement and a 2-s orbital mixing step performed at 250 rpm before commencing the measurements. The resulting polymerization curves were normalized and plotted in GraphPad Prism 8.0.0.

### Electron microscopy

*Pf*ActI wild-type and mutant samples were polymerized for 16 h at 298 K at a concentration of 20 μM in F buffer. Prior to application on carbon-coated 200-mesh Cu grids (Electron Microscopy Sciences, Hatfield, PA), samples were diluted to a final concentration of 1 μM and immediately applied on the grids. Samples were incubated for 60 s on the grids, dried from the side using prewetted Whatman paper, and washed with 3 drops of F buffer. Then, they were stained with 2% uranyl acetate, first for 2 s and then for 60 s, in a fresh drop before drying from the side as before and then drying in air. The grids were imaged using a JEOL JEM-1230 microscope (JEOL Ltd., Tokyo, Japan) operated at 80 kV and with a final pixel size of 1.22 nm. The images were analyzed using the ridge detection plugin available in ImageJ 2.0.0 [58]. The calculated length measurement for any given filament corresponds to the portion that lies within a given frame and therefore cannot be used as a measure of the actual length of the long filaments.

### Protein crystallization

*Pf*ActI-G1 and *Pb*ActII-G1 complexes in the Mg state were prepared essentially as described by Panneerselvam and colleagues [27], with the exception that CdCl_2_ was replaced by 1 mM MgCl_2_. In some cases, crystals were grown by streak seeding as described by Panneerselvam and colleagues [27], and in others, crystals were obtained directly from optimization screens without seeding. Cryoprotection was achieved by soaking for 5 to 30 s using the same condition as for the crystallization with a higher precipitant concentration (PEG3350, 22%–28%) and PEG400 at 10% to 20% as the cryoprotectant. Protein buffer components were also included in the cryosolutions at concentrations of 1 mM MgCl_2_, 0.5 mM ADP, and 0.5 mM TCEP for the Mg conditions and 0.2 mM CaCl_2_, 0.5 mM ATP, and 0.5 mM TCEP for the Ca conditions. The pH of the crystallization reservoir buffer (0.1 M Bis-Tris) varied from 5.8 to 6.5. Mg-ADP-*Pf*ActI-G1 crystals were cryoprotected in a solution containing 50 mM potassium phosphate. Mg-ADP-AlF_n_-*Pf*ActI(F54Y)-G1 crystals were prepared by adding a solution of 20% PEG3350, 0.1 M Bis-Tris (pH 6.0), 0.2 M KSCN, and 1 mM AlF_n_ solution directly into the drops and incubated for a few minutes before cryoprotection with a solution as described above. The AlF_n_ solution consisted of premixed AlCl_3_ and NaF in a 1:4 molar ratio. The minimum time between data collection from a crystal yielding structures with ATP/ADP mixtures and ADP only was 6 mo for Mg-*Pf*ActI-F54Y crystals, whereas the time from crystallization to data collection from Mg-*Pb*ActII was only 2 wk.

### Diffraction data collection, processing, and structure refinement

Crystallization data were collected at 100 K at several beamlines. Mg-ATP/ADP-*Pf*ActI, Mg-ADP-P_i_-*Pf*ActI, Mg-ADP-F54Y, and Mg-*Pb*ActII were collected at beamline P13 of PETRA III, DESY (Hamburg, Germany); Ca-F54Y and Mg-AlF_n_-F54Y were collected at I24 of Diamond Light Source (Didcot, UK); Mg-F54Y, Mg-H74Q, and Mg-A272W were collected at I04-1 of Diamond Light Source (Didcot, UK), Ca-G115A was collected at ID23-1 of ESRF (Grenoble, France); and Mg-G115A was collected at MX-14.1 of BESSY (Berlin, Germany). Diffraction images were processed using the XDS package [59]. Structure determination and refinement were carried out using programs of the PHENIX suite [60]. Initial phases were found by molecular replacement with PHASER [61], using the Ca-ATP-*Pf*ActI-G1 structure (PDB ID 4CBU) as the search model for the *Pf*ActI structures and the Ca-ATP-*Pb*ActI-G1 structure (PDB ID 4CBX) for the *Pb*ActII structure. Additionally, MR-SAD using Autosol [62] was used to reduce model bias in the Mg-ADP-AlF_n_-F54Y structure. Structure refinement was carried out using phenix.refine [63].

### PCA

There are 2 main structural rearrangements recognized in actin: the twistedness of the 2 main domains (SD1–2 and SD3–4) along an axis that pierces SD1 and SD3 at their respective centers and the openness of the nucleotide-binding cleft as a rotation around an axis perpendicular to the twist axis and to the plane of the F-actin monomer. We analyzed 147 unique actin structures found in the PDB using BIO3D [64] at resolution ≤4 Å, together with the structures reported here, and found that these movements are captured well by PCA in PC1 (twistedness) and PC2 (openness) that contain 78% of total variance (S1 Movie). All actin sequences were first aligned using MUSCLE [65], and the sequence gaps were removed. Then, the structures were aligned on a common core before PCA analysis [66]. The common core was determined by iterative pruning of atoms until a preset volume threshold of 0.5 was reached [64]. In structures with multiple chains of actins, all chains were used for the PCA analysis, but a single chain was used for plotting.

In a plot of PC1 versus PC2 (S4 Fig), most actin structures cluster at the center of the plot. This large cluster contains all structures reported in this study as well as structures of actin bound to ABPs such as gelsolin and profilin. Several outliers to this large cluster form their own distinct groups. Filament structures cluster at low twistedness and average openness, open profilin-actin structures cluster at high openness and average twistedness, free G-actin structures cluster at high twistedness and average openness, and finally, ADF/cofilin bound actin structures cluster at high twistedness and low openness. We also analyzed *Plasmodium* actin structures as their own set by similar PCA analysis. PC1 and PC2 contain 84% of total variance in this data set, and their trajectories are toned-down versions of the twistedness and openness of the full data set (S1 Movie). Although PC1 in the limited data set can easily be recognized as the twisting motion of the full data set (due to the presence of the F-*Pf*ActI model), the opening-closing motion is slightly ambiguous (due to the lack of an open *Pf*ActI model) and is therefore indicated with an asterisk.

### Domain motion analysis

To support the PCA analysis, we measured 3 parameters of 4 sets of structures: the domain distance between SD2 and SD4 (d_2–4_), the phosphate clamp distance (b_2_ [24]), and the torsion angle defined by all 4 SDs (θ). The d_2–4_ distance was measured by a distance between the mass centers of the Cα atoms of residues 35–39 and 52–73 for SD2 and 183–269 for SD4. The phosphate clamp distance was measured as the distance between α-carbons of Gly16 and Asp158. The torsion angle θ was measured using the mass centers of α-carbons from all 4 domains using the residue assignment defined above for SD2 and SD4, as well as residues 6–32, 77–137, and 340–366 for SD1 and residues 140–182 and 270–337 for SD3. The models used were (i) wild-type *Pf*ActI structures in the Ca-ATP, Mg-ATP/ADP, Mg-ADP, and F-ADP states; (ii) *Pf*ActI F54Y structures in Ca-ATP, Mg-ADP-AlF_3_, and Mg-ADP; (iii) *Pb*ActII structures in Ca-ATP and Mg-ADP states; and (iv) *D*. *discoideum* actin structures of mutant E205A/R206A/E207A/P109I in Ca-ATP and Mg-ATP and mutant E205A/R206A/E207A/P109A in Ca-ATP and Mg-ADP. The results are presented in S7 Table. For the *D*. *discoideum* structures, all residue assignments are −1 relative to the numbers presented above. For *Pb*ActII, residue assignments for residue numbers smaller than 232 were −1 relative to *Pf*ActI and others as for *Pf*ActI.

### Analysis of ID–OD interaction energies

The ID–OD contacts were analyzed using the Amino Acid Interaction (INTAA) server [67] that uses point-charge electrostatics and Lennard-Jones potentials to calculate an interaction energy term between selected amino acids. Three key residues from the ID (Arg178, Asp180, and Arg184) were selected, and interaction energies were calculated for 2 patches on the OD side (Lys69-Ile76 of the H loop and Glu108-Lys114) for the 5 models presented in Fig 7. Individual models were generated by selecting conformations with the least number of clashes in the area of interest. Hydrogen atoms were then added to these models using the AddH module in Chimera [68], resulting in singly protonated His74 at the δN. In the case of the F-*Pf*ActI model in conformation 2a (IV in Fig 7), the original F-actin model was superposed with the 2a conformation model derived from the Mg-ATP/ADP structure using SD3 only. The coordinates of the A loop were then transferred from the Mg-ATP/ADP model to the F-actin model, and the model was subsequently energy minimized in UCSF Chimera. One hundred steps of steepest descent minimization with 0.02-Å step size was followed by 10 steps of conjugate gradient minimization with 0.02-Å step size and keeping all the atoms except the A loop static, which kept in line with the hypothesis that the loop movement is the singular event affecting the ID–OD stability.

## Supporting information

S1 Table P_i_ release rates reported in the literature. P_i_, inorganic phosphate.
(DOCX)

S2 Table Phosphate release rates of actins in Ca, Mg, and MgK conditions and activation by Mg^2+^ and K^+^ at actin concentrations of 1 μM. (DOCX)

S3 Table Phosphate release rates of actins in Ca, Mg, and MgK conditions and activation by Mg^2+^ and K^+^ at actin concentrations of 3 to 6 μM. (DOCX)

S4 Table Crystallization conditions for the structures reported in this study. (DOCX)

S5 Table Crystallization states, ATP occupancies, pH, and resolution. (DOCX)

S6 Table Crystallographic data collection and refinement statistics. (DOCX)

S7 Table Distances and angles describing the SD positions from 4 sets of structures. SD, subdomain.
(DOCX)

S1 Fig P_i_ release as a function of time from high concentrations of *Pf*ActI, *Pb*ActII, and α-actin. P_i_ release curves of (A) *Pf*ActI at 3.5 μM, (B) *Pb*ActII at 3.8 μM, and (C) α-actin at 5.9 μM in Ca-ATP (dashed black line), Mg-ATP (black line) and MgK (red line) conditions. The underlying data for this figure can be found in S4 Data. *Pb*ActII, *Plasmodium berghei* actin II; *Pf*ActI, *P*. *falciparum* actin I; P_i_, inorganic phosphate.
(TIF)

S2 Fig Alternate refinements of the active site of Mg-ATP/ADP-*Pf*ActI and identification of mHis73 in *Pb*ActII. Presence of ATP/ADP mixture and K in the active site of *Pf*ActI and the presence of mHis73 in *Pb*ActII evidenced by electron density maps and mass spectrometry. Difference density maps (mF_o_-dF_c_) at 4 σ of *Pf*ActI active site refined with (A) ADP only, (B) ATP only, and (C) K replaced by full-occupancy water. (D) Electron density (2mF_o_-dF_c_) and difference density (mF_o_-dF_c_) maps around His73 at 1 σ and 4 σ, respectively, with a model refined as methylated and unmethylated His73. (E) Mass spectrum of full-length *Pb*ActII expressed in Sf21 insect cells. Spectrum measured by ESI-LCMS from 12 μM *Pb*ActII with 1.45% (v/v) TFA. The 42,822-Da peak corresponds to methylated *Pb*ActII, whereas the theoretical average molecular weight for unmethylated *Pb*ActII is 42,809 Da (Δm = 13 Da). The peak at 42,805 Da is unknown but could result from, e.g., intramolecular disulfides (2 × −2 Da). The underlying data for this figure can be found in S5 Data. ESI-LCMS, electrospray ionization liquid chromatography mass spectrometry; mHis73, methylated His73; *Pb*ActII, *Plasmodium berghei* actin II; *Pf*ActI, *P*. *falciparum* actin I; Sf21, *Spodoptera frugiperda* cell line; TFA, trifluoroacetic acid.
(TIF)

S3 Fig Delayed P_i_ release in K270M and A272W mutants of *Pf*ActI and pH dependence of P_i_ release in wild-type *Pf*ActI. (A) The effect of pH on P_i_ release by *Pf*ActI. The P_i_ release rate was measured in Mg-ATP conditions at a range of pH values. The rates were normalized relative to the sample at pH 7.34, which corresponds to the standard assay conditions. (B) Delayed phosphate release in mutants K270M and A272W of *Pf*ActI. P_i_ release curves of *Pf*ActI wild-type (3.5 μM), K270M (6.4 μM), and A272W (4.3 μM) mutants in Mg-ATP (black lines) and MgK (red lines) conditions. The inset shows the complete curves with the same units as the main plot. The curves have been translated in Y to improve clarity. The underlying data for this figure can be found in S6 Data. *Pf*ActI, *Plasmodium falciparum* actin I; P_i_, inorganic phosphate.
(TIF)

S4 Fig PCA of actin structures in the PDB and from this study. (A) Plot of twistedness (PC1) versus openness (PC2) of the full data set of 147 actin structures (see also S1 Movie). Defined structural groups of filament structures (dark purple), Pfn-bound open structures (orange), free G actin structures (light purple), and ADF/cofilin-bound structures (pink) are indicated with F, Pfn, G, and C, respectively. The large heterogeneous group in the middle is shaded in gray. Structures of interest are indicated with circles or squares and names or PDB identifiers, whereas others are indicated with black dots. (B) Zoomed view of (A) containing the *Plasmodium* actin structures (excluding *Pb*ActII Mg-ADP) as well as 4 mutant *D*. *discoideum* actin structures [16] constituting a full set of nucleotide and divalent cation states. (C) PCA of *Plasmodium* actin structures only, with similar notation as in (A). (D) Zoomed view of (C) containing all relevant *Pf*ActI structures excluding the H74Q mutant and the *Pb*ActI–α-actin chimera [9]. The lines and dashed lines between the *Pf*ActI and *Pb*ActII structures indicate the path in the hydrolytic direction (ATP–ATP/ADP–ADP) as appropriate for each set of structures. F structures are underlined, all other structures are of the G form. The underlying data for this figure can be found in S7 Data. ADF, actin depolymerizing factor; C, ADF/cofilin-bound structures; F, filamentous; G, globular; *Pb*ActII, *Plasmodium berghei* actin II; PC, principal component; PCA, principal component analysis; PDB, Protein Data Bank; *Pf*ActI, *P*. *falciparum* actin I; Pfn, profilin.
(TIF)

S5 Fig Residue-level anisotropic B factors of *Pf*ActI in Ca-ATP, Mg-ATP/ADP, and Mg-ADP states. Anisotropic B factors show relative destabilization of SD2 of *Pf*ActI during ATP hydrolysis. The size of the ellipsoids is proportional to residue-level B factors and the shape to the direction of anisotropy. Presence of Mg induces an opening of the interdomain cleft and a directional destabilization of SD2 along the rotation of the G–F transition axis (through the mass centers of SD1 and SD3). (Front) View from the side of nucleotide entry (ID on the left, OD on the right). (Back) View from the opposite side of nucleotide entry. (OD) Side view from the OD side with ID faded out. (ID) Side view from the ID side with OD faded out. Numbers 1 through 4 indicate the respective SDs, and the arrows indicate the main direction of anisotropy. F, filamentous; G, globular; ID, inner domain; OD, outer domain; *Pf*ActI, *Plasmodium falciparum* actin I; SD, subdomain.
(TIF)

S6 Fig Backbone conformations of residues around His74 in *Pf*ActI and His73 in *Pb*ActII. Orientations of the H-loop Glu73-His74 backbone in *Pf*ActI wild type and mutants A272W and H74Q as well as those of Glu72-His73 backbone in *Pb*ActII. *Pb*ActII, *Plasmodium berghei* actin II; *Pf*ActI, *P*. *falciparum* actin I.
(TIF)

S7 Fig Multiple sequence alignment of actins from various kingdoms of life. Sequence alignment of selected actin amino acid sequences with secondary structure elements from *Pf*ActI [9]. α-helices and β-strands are indicated with helical symbols and arrows as well as Greek letters and numbers, whereas 3_10_ helices are only indicated by helical symbols. The loops and the plug region discussed in the text are highlighted under the sequences. The sequences are divided into 2 groups: (1) apicomplexan actins, including *Pf*ActI (Q8I4X0), *Pb*ActII (Q4YU79), and *Tg*Act (P53476) and (2) canonical actins from *Dd*Act (P07830), *Sc*Act (P60010), *At*Act (P0CJ46), *Hs*Act_alpha_sk (P68133), *Hs*Act_beta (P60709), *Hs*Act_gamma (P63261). Human actins are identical in sequence to corresponding other mammalian actins. Arrowheads indicate mutations studied in this work. The sequence numbering corresponds to that of *Pf*ActI. *At*Act, *Arabidopsis thaliana* actin; *Dd*Act, *Dictyostelium discoideum* actin; *Hs*Act_alpha_sk, *Homo sapiens* skeletal muscle α actin; *Hs*Act_beta, *H*. *sapiens* β actin; *Hs*Act_gamma, *H*. *sapiens* γ actin; *Pb*ActII, *Plasmodium berghei* actin II; *Pf*ActI, *P*. *falciparum* actin I; *Sc*Act, *Saccharomyces cerevisiae* actin; *Tg*Act, *Toxoplasma gondii* actin.
(TIF)

S8 Fig Atomic-level B factors in the A loop of different *Plasmodium* actin structures. Temperature factors of protein side-chain atoms in the vicinity of the A loop. *Pf*ActI in (A) Ca-ATP state (all conformers), Mg-ATP/ADP state conformers A, B, and C (B–D), Mg-ADP state conformers A and B (E, F). *Pb*ActII in Ca-ATP state (G) and Mg-ADP state (H). The size of the sphere relates to the maximum and minimum B factors of each structure, whereas the color relates to the first quartile (Q1, dark cyan), median (Q2, white), and third quartile (Q3, maroon) of the total distribution of B factors in each structure. *Pb*ActII, *Plasmodium berghei* actin II; *Pf*ActI, *P*. *falciparum* actin I.
(TIF)

S9 Fig Interactions of Arg178 of ID with the P loop of OD. Connections formed by Arg178 between the P loop of SD1 and the H loop of SD2 in the *Pf*ActI Mg-ATP/ADP structure. (A) Arg178 forms hydrogen bonds with backbone O of Pro110 in SD1 and His74 in SD2 in conformation 1b. (B) Interaction between Arg178 and SD2 is maintained by the cation-π interaction via His74 in conformation 2a, but the connection to SD1 is lost. (C) Contacts between the ID and OD are preserved in the F-*Pf*ActI model that is also in conformation 1b. OD residues are indicated in orange carbon atoms, whereas ID residues are indicated in gray. F, filamentous; ID, inner domain; OD, outer domain; *Pf*ActI, *Plasmodium falciparum* actin I; SD, subdomain.
(TIF)

S10 Fig Plot of residue-level B factors in *Pf*ActI and *Pb*ActII. Plot of residue-level B factors of *Pf*ActI in (gray line) Ca-ATP, (blue line) Mg-ATP/ADP and (black line) Mg-ADP states as well as *Pb*ActII in (brown line) Ca-ATP and (orange line) Mg-ADP states. The SDs are annotated as SD1 (yellow), SD2 (red), SD3 (cyan), and SD4 (magenta). The approximate site of residues 61–66 that are built into weak density is marked by a black arrow. The underlying data for this figure can be found in S8 Data. *Pb*ActII, *Plasmodium berghei* actin II; *Pf*ActI, *P*. *falciparum* actin I, SD, subdomain.
(TIF)

S1 Movie Principal component trajectories of Cα traces from PCA of actins. PC1 (twistedness) and PC2 (openness) are shown for the full data set of 147 actin chains and for the limited data set comprising only *Plasmodium* actin structures. PC, principal component; PCA, principal component analysis.
(MP4)

S2 Movie Interpolated trajectories of the models used in Fig 7 to generate a comprehensive view of conformational changes taking place in *Pf*ActI during the polymerization and fragmentation processes. *Pf*ActI, *Plasmodium falciparum* actin I.
(MP4)

S1 Data The underlying raw data for Fig 1A. (XLSX)

S2 Data The underlying raw data for Fig 4B. (XLSX)

S3 Data The underlying raw data for Fig 7. (XLSX)

S4 Data The underlying raw data for S1 Fig. (XLSX)

S5 Data The underlying raw data for S2 Fig. (XLSX)

S6 Data The underlying raw data for S3 Fig. (XLSX)

S7 Data The underlying raw data for S4 Fig. (XLSX)

S8 Data The underlying raw data for S10 Fig. (XLSX)

## Abbreviations

EGTA: ethylene glycol-bis[2-aminoethyl ether]-N,N,N’,N’-tetraacetic acid
F: filamentous
G: globular
Hsc70: Heat shock cognate 71 kDa protein
ID: inner domain
MDCC-PBP: 7-diethylamino-3-[N-(2-maleimidoethyl)carbamoyl]coumarin-labeled phosphate-binding protein
mHis73: methylated His73
OD: outer domain
*Pb*ActII: *Plasmodium berghei* actin II
PC: principal component
PCA: principal component analysis
PDB: Protein Data Bank
*Pf*ActI: *P*. *falciparum* actin I
P_i_: inorganic phosphate
RMSD: root-mean-square deviation
*Sc*Act: *Saccharomyces cerevisiae* actin
SD: subdomain
TEV: tobacco etch virus
*Tg*Act: *Toxoplasma gondii* actin
