# Supplementary material for: Atomic view into Plasmodium actin polymerization, ATP hydrolysis, and fragmentation
Source: PLoS Biol. 2019 Jun 14;17(6):e3000315. doi: 10.1371/journal.pbio.3000315 (PMC6599135; doi:10.1371/journal.pbio.3000315)
Supplement: S1 Table — Pi, inorganic phosphate. (DOCX) [file pbio.3000315.s001.docx]

**S1 Table**: Reported P_i_ release rates in the literature.

|  | **Condition** | **Actin**  (µM) | **Rate**  (10^-4^s^-1^) | **State** | **Reference** |
| --- | --- | --- | --- | --- | --- |
| α-actin | F-buffer | 10 | 0.15-0.26 | eq | 20 |
| α-actin | Ca / Mg | 11.5 / 7.6 | 0.23 / 0.47 | eq_Ca_ / eq_Mg_ | 9 |
| *Pf*ActI | Ca / Mg | 8.2 / 8.5 | 1.3 / 3.1 | eq_Ca_ / eq_Mg_ | 9 |
| *Pb*ActII | Ca / Mg | 3.9 / 7.6 | 1.3 / 1.9 | eq_Ca_ / eq_Mg_ | 9 |
| AP-actin | Mg | 95 | 0.07 | m | 30 |

eq: equilibrium

m: monomeric
