# Supplementary material for: Atomic view into Plasmodium actin polymerization, ATP hydrolysis, and fragmentation
Source: PLoS Biol. 2019 Jun 14;17(6):e3000315. doi: 10.1371/journal.pbio.3000315 (PMC6599135; doi:10.1371/journal.pbio.3000315)
Supplement: S2 Table — (DOCX) [file pbio.3000315.s002.docx]

**S2 Table:** Phosphate release rates of actins in Ca, Mg and MgK conditions and activation by Mg^2+^ and K^+^ at actin concentrations of 1 µM.

|  | **Condition** | | |  | **Activation** | |
| --- | --- | --- | --- | --- | --- | --- |
|  | **Ca**  (10^-4^ s^-1^) | **Mg**  (10^-4^ s^-1^) | **MgK**  (10^-4^ s^-1^) |  | by **Mg^2+^**  (Mg/Ca) | by **K^+^**  (MgK/Mg) |
| K270M^†^ | 0.21±0.01^*^ | 4.6±0.19^*^ | 6.2±0.60^*^ |  | 22±1.7^*^ | 1.4±0.2^*^ |
| A272W^†^ | 0.52±0.02^*^ | 9.78±0.06^*^ | 9.9±0.19^*^ |  | 18.9±0.9^*^ | 1.02±0.03^*^* |
| A272C^†^ | 0.30±0.01^*^ | 1.54±0.06 | 1.69±0.06^*^ |  | 5.1±0.4^*^ | 1.09±0.08^*^* |
| E49G^†^ | 0.58±0.02^*^ | 2.8±0.13^*^ | 2.8±0.20^*^ |  | 4.7±0.4^*^ | 1.0±0.1 |
| F54Y^†^ | 1.20±0.08^*^ | 3.14±0.09^*^ | 2.8±0.12^*^ |  | 2.6±0.3 | 0.90±0.07 |
| P42Q/E49G^†^ | 0.52±0.03^*^ | 1.18±0.04^*^ | 1.18±0.09 |  | 2.3±0.2 | 1.0±0.1 |
| *Pf*Act1 *wt* | 0.74±0.03 | 1.62±0.05 | 1.3±0.12 |  | 2.2±0.2 | 0.8±0.1 |
| G115A^†^ | 0.70±0.07 | 1.05±0.04^*^ | 1.00±0.07 |  | 1.6±0.6 | 1.0±0.2 |
| P42Q^†^ | 1.24±0.03^*^ | 1.75±0.02 | 1.52±0.09 |  | 1.41±0.05^*^ | 0.87±0.07 |
| H74Q^†^ | 0.27±0.03^*^ | 0.27±0.01^*^ | 0.21±0.03^*^ |  | 1.0±0.2^*^ | 0.8±0.2 |
| *Pb*ActII | 0.60±0.04^*^ | 0.67±0.05^*^ | 0.56±0.02^*^ |  | 1.1±0.1^*^ | 0.83±0.09 |
| α-actin | 0.22±0.01^*^ | 0.68±0.01^*^ | 1.99±0.02^*^ |  | 3.1±0.2^*^ | 2.92±0.08^*^ |

*: P < 0.01 (N = 3), two-tailed Student’s T-test vs. corresponding values of *Pf*ActI wildtype.

†: Mutants are of *Pf*ActI.

*Pf*ActI results are ordered by Mg^2+^ activation.

Errors represent standard deviations.
