# Supplementary material for: Atomic view into Plasmodium actin polymerization, ATP hydrolysis, and fragmentation
Source: PLoS Biol. 2019 Jun 14;17(6):e3000315. doi: 10.1371/journal.pbio.3000315 (PMC6599135; doi:10.1371/journal.pbio.3000315)
Supplement: S3 Table — (DOCX) [file pbio.3000315.s003.docx]

**S3 Table.**  Phosphate release rates of actins in Ca, Mg and MgK conditions and activation by Mg^2+^ and K^+^ at actin concentrations of 3-6 µM.

|  | **Condition** | | |  | **Activation** | |
| --- | --- | --- | --- | --- | --- | --- |
|  | **Ca**  (10^-4^ s^-1^) | **Mg**  (10^-4^ s^-1^) | **MgK**  (10^-4^ s^-1^) |  | by **Mg^2+^**  (Mg/Ca) | by **K^+^**  (MgK/Mg) |
| *Pf*ActI  (3.5 µM) | 0.38±0.004 | 1.7±0.04 | 1.7±0.08 |  | 4.5±0.2 | 1.0±0.08 |
| *Pb*ActII  (3.8 µM) | 0.15±0.009 | 1.6±0.03 | 7.0±0.4 |  | 11.0±1.0 | 5.0±0.4 |
| α-actin  (5.9 µM) | 0.095±0.003 | 2.9±0.06 | 7.0±0.1 |  | 31.0±1.6 | 2.2±0.09 |

P < 0.01 (N = 3) for all *Pb*ActII and α-actin values, two-tailed Student’s T-test vs. corresponding values of *Pf*ActI wildtype.

Errors represent standard deviations.
