# Supplementary material for: Atomic view into Plasmodium actin polymerization, ATP hydrolysis, and fragmentation
Source: PLoS Biol. 2019 Jun 14;17(6):e3000315. doi: 10.1371/journal.pbio.3000315 (PMC6599135; doi:10.1371/journal.pbio.3000315)
Supplement: S4 Table — (DOCX) [file pbio.3000315.s004.docx]

**S4 Table.** Crystallization conditions for the structures reported in this study.

|  | **State** | **PEG3350**  **(%)** | **K-SCN**  **(M)** | **pH*** | **Protein in drop**  **(mg/ml)** | **Seeded** |
| --- | --- | --- | --- | --- | --- | --- |
| *Pf*ActI wt | Mg-ATP/ADP | 23 | 0.2 | 6.0 | 4.0 | no |
|  | Mg-ADP | 23 | 0.2 | 6.2 | 4.0 | no |
| *Pb*ActII wt | Mg-ADP | 23 | 0.2 | 6.3 | 4.0 | no |
| F54Y | Ca-ATP | 23 | 0.2 | 5.9 | 4.0 | no |
|  | Mg-ADP-AlF_n_^†^ | 23 | 0.2 | 5.9 | 4.0 | no |
|  | Mg-ADP | 23 | 0.2 | 5.9 | 4.0 | no |
| G115A | Ca-ATP | 23 | 0.2 | 5.9 | 4.0 | no |
|  | Mg-ATP/ADP | 23 | 0.2 | 5.9 | 4.0 | no |
| H74Q | Mg-ATP | 11 | 0.2 | 5.8 | 4.0 | yes |
| A272W | Mg-ATP/ADP | 11 | 0.2 | 5.8 | 4.0 | yes |

^*^0.1 M Bis-Tris

^†^Crystallized with AlF_3_
