# Supplementary material for: Atomic view into Plasmodium actin polymerization, ATP hydrolysis, and fragmentation
Source: PLoS Biol. 2019 Jun 14;17(6):e3000315. doi: 10.1371/journal.pbio.3000315 (PMC6599135; doi:10.1371/journal.pbio.3000315)
Supplement: S5 Table — (DOCX) [file pbio.3000315.s005.docx]

**S5 Table.** Crystallization states, ATP occupancies, pH and resolution.

|  | **Divalent Cation** | **Nucleotide State** | **ATP Occ.** | **pH** | **Resolution**  (Å) | **PDB ID** |
| --- | --- | --- | --- | --- | --- | --- |
| *Pf*ActI wt | Ca^*^ | ATP^*^ | 1.00^*^ | 6.5 | 1.30^*^ | 4CBU^*^ |
|  | Mg | ATP/ADP | 0.79 | 6.0 | 1.24 | 6I4D |
|  | Mg | ADP | 0 | 6.2 | 1.22 | 6I4E |
| *Pb*ActII wt | Ca^*^ | ATP^*^ | 1.00^*^ | 6.5 | 2.20^*^ | 4CBX^*^ |
|  | Mg | ADP | 0 | 6.3 | 1.87 | 6I4M |
| F54Y | Ca | ATP | 1.00 | 5.9 | 1.40 | 6I4H |
|  | Mg^†^ | ATP/ADP^†^ | 0^†^ | 5.9 | 1.90^†^ | 6I4I |
|  | Mg | ADP | 0 | 5.9 | 1.50 | 6I4J |
| G115A | Ca | ATP | 0 | 5.9 | 1.83 | 6I4K |
|  | Mg | ATP/ADP | 0.54 | 5.9 | 1.83 | 6I4L |
| H74Q | Mg | ATP | 0 | 5.8 | 2.00 | 6I4G |
| A272W | Mg | ATP/ADP | 0.32 | 5.8 | 1.50 | 6I4F |

*Structures reported in ref. 9.

^†^Crystallized with AlF_3_
