# Supplementary material for: Atomic view into Plasmodium actin polymerization, ATP hydrolysis, and fragmentation
Source: PLoS Biol. 2019 Jun 14;17(6):e3000315. doi: 10.1371/journal.pbio.3000315 (PMC6599135; doi:10.1371/journal.pbio.3000315)
Supplement: S6 Table — (DOCX) [file pbio.3000315.s006.docx]

**S6 Table.** Crystallographic data collection and refinement statistics.

|  | *Pf*ActI | | | | *Pb*ActII |
| --- | --- | --- | --- | --- | --- |
|  | Mg-ATP/ADP  (PDB 6I4D) | Mg-ADP  (PDB 6I4E) | Mg-ATP/ADP A272W  (PDB 6I4F) | Mg-ATP  H74Q  (PDB 6I4G) | Mg-ADP  (PDB 6I4M) |
| **Data collection** |  |  |  |  |  |
| Wavelength (Å) | 1.031 | 0.954 | 0.916 | 0.916 | 1.031 |
| Space group | P2_1_22_1_ | P2_1_22_1_ | P2_1_22_1_ | P2_1_2_1_2_1_ | P2_1_ |
| Cell dimensions |  |  |  |  |  |
| a, b, c (Å) | 68.8, 71.5, 110.0 | 68.5, 71.3, 110.0 | 68.8, 71.5, 110.0 | 68.6, 110.6, 138.8 | 61.4, 61.5, 64.9 |
| α, β, γ (°) | 90.0, 90.0, 90.0 | 90.0, 90.0, 90.0 | 90.0, 90.0, 90.0 | 90.0, 90.0, 90.0 | 90.0, 92.5, 90.0 |
| Resolution (Å) | 59.9-1.24  (1.28-1.24) | 49.4-1.22  (1.26-1.22) | 45.2 -1.50  (1.55 - 1.50) | 48.8-2.00  (2.07-2.00) | 64.9-1.87  (1.94-1.87) |
| R_merge_ | 0.058 (0.990) | 0.047 (1.129) | 0.095 (2.123) | 0.166 (1.851) | 0.038 (0.296) |
| I / σ(I) | 12.5 (1.0) | 16.3 (1.5) | 10.7 (0.8) | 5.6 (0.69) | 15.1 (2.4) |
| CC_1/2_ | 0.999 (0.385) | 0.999 (0.513) | 0.999 (0.365) | 0.994 (0.437) | 0.999 (0.826) |
| Completeness (%) | 98.8 (88.2) | 99.8 (99.8) | 100.0 (100.0) | 95.6 (92.8) | 96.8 (82.5) |
| Redundancy | 3.7 (2.7) | 5.4 (5.4) | 6.7 (6.7) | 5.3 (4.3) | 1.9 (1.7) |
| **Refinement** |  |  |  |  |  |
| Resolution (Å) | 59.9-1.24  (1.28-1.24) | 49.4-1.22  (1.26-1.22) | 45.2 -1.50  (1.55 -1.50) | 48.8-2.00  (2.07-2.00) | 64.9-1.87  (1.94-1.87) |
| No. of reflections | 152,395 | 159,860 | 167,756 | 131,940 | 73,246 |
| R_work_ / R_free_ | 0.130 / 0.155 | 0.136 / 0.156 | 0.156 / 0.192 | 0.222 / 0.267 | 0.143 / 0.179 |
| No. of atoms |  |  |  |  |  |
| Protein | 4,738 | 4,427 | 3,993 | 7,582 | 3,958 |
| Ligands | 83 | 66 | 99 | 85 | 36 |
| Solvent | 784 | 484 | 521 | 399 | 446 |
| B-factors |  |  |  |  |  |
| Protein | 16.3 | 21.6 | 33.0 | 45.8 | 28.4 |
| Ligands | 18.3 | 40.0 | 48.5 | 43.9 | 19.2 |
| Solvent | 28.6 | 31.9 | 42.8 | 43.1 | 35.7 |
| R.m.s. deviations |  |  |  |  |  |
| Bond lengths (Å) | 0.011 | 0.012 | 0.011 | 0.008 | 0.012 |
| Bond angles (°) | 1.5 | 1.5 | 1.5 | 1.2 | 1.4 |

Values in parentheses are for the highest-resolution shell.

Friedel pairs are considered separate reflections.

**S6 Table.** Crystallographic data collection and refinement statistics. (*continued*)

|  | *Pf*ActI | | | | |
| --- | --- | --- | --- | --- | --- |
|  | Ca-ATP  F54Y  (PDB 6I4H) | Mg-ATP/ADP^†^  F54Y  (PDB 6I4I) | Mg-ADP  F54Y  (PDB 6I4J) | Ca-ATP  G115A  (PDB 6I4K) | Mg-ATP/ADP  G115A  (PDB 6I4L) |
| **Data collection** |  |  |  |  |  |
| Wavelength (Å) | 0.969 | 0.969 | 1.137 | 0.954 | 0.918 |
| Space group | P2_1_22_1_ | P2_1_22_1_ | P2_1_22_1_ | P2_1_22_1_ | P2_1_22_1_ |
| Cell dimensions |  |  |  |  |  |
| a, b, c (Å) | 68.87, 71.54, 110.6 | 68.61, 71.33, 109.25 | 68.56, 71.35, 109.67 | 69.83, 71.38, 110.93 | 69.45, 71.33, 110.31 |
| α, β, γ (°) | 90.0, 90.0, 90.0 | 90.0, 90.0, 90.0 | 90.0, 90.0, 90.0 | 90.0, 90.0, 90.0 | 90.0, 90.0, 90.0 |
| Resolution (Å) | 43.8-1.40  (1.45-1.40) | 45.1-1.90  (1.97-1.90) | 45.1-1.50  (1.55-1.50) | 43.8-1.83  (1.90-1.83) | 45.4-1.83  (1.90-1.83) |
| R_merge_ | 0.050 (0.98) | 0.096 (0.710) | 0.070 (0.938) | 0.136 (0.989) | 0.129 (1.054) |
| I / σ(I) | 12.2 (1.1) | 9.1 (1.8) | 7.8 (0.76) | 8.3 (0.9) | 5.0 (0.7) |
| CC_1/2_ | 0.998 (0.398) | 0.995 (0.591) | 0.999 (0.401) | 0.993 (0.373) | 0.990 (0.327) |
| Completeness (%) | 99.5 (99.0) | 99.5 (99.1) | 97.4 (95.1) | 99.8 (98.5) | 95.9 (96.2) |
| Redundancy | 3.3 (3.3) | 3.3 (3.3) | 2.7 (2.4) | 3.7 (2.6) | 2.0 (2.0) |
| **Refinement** |  |  |  |  |  |
| Resolution (Å) | 43.8-1.40  (1.45-1.40) | 45.1-1.90  (1.97-1.90) | 45.1-1.50  (1.55-1.50) | 43.8-1.83  (1.90-1.83) | 45.4-1.83  (1.90-1.83) |
| No. of reflections | 206,784 | 81,203 | 162,223 | 94,309 | 89,595 |
| R_work_ / R_free_ | 0.143 / 0.163 | 0.179 / 0.217 | 0.165 / 0.198 | 0.159 / 0.197 | 0.200 / 0.243 |
| No. of atoms |  |  |  |  |  |
| Protein | 4,156 | 3,893 | 4,123 | 4,117 | 3,826 |
| Ligands | 42 | 39 | 77 | 66 | 67 |
| Solvent | 694 | 276 | 531 | 527 | 383 |
| B-factors |  |  |  |  |  |
| Protein | 24.1 | 36.7 | 28.2 | 26.2 | 28.7 |
| Ligands | 18.9 | 34.0 | 30.5 | 35.3 | 23.4 |
| Solvent | 37.2 | 39.0 | 37.7 | 35.6 | 32.3 |
| R.m.s. deviations |  |  |  |  |  |
| Bond lengths (Å) | 0.009 | 0.008 | 0.014 | 0.007 | 0.009 |
| Bond angles (°) | 1.4 | 1.2 | 1.5 | 1.3 | 1.3 |

Values in parentheses are for the highest-resolution shell.

Friedel pairs are considered separate reflections.

†Crystallized in the presence of AlF_n_.
