# Supplementary material for: Atomic view into Plasmodium actin polymerization, ATP hydrolysis, and fragmentation
Source: PLoS Biol. 2019 Jun 14;17(6):e3000315. doi: 10.1371/journal.pbio.3000315 (PMC6599135; doi:10.1371/journal.pbio.3000315)
Supplement: S7 Table — SD, subdomain. (DOCX) [file pbio.3000315.s007.docx]

**S7 Table.** Distances and angles describing the subdomain positions from four sets of structures.

|  | *Pf*ActI wt | | | *Pf*ActI F54Y | | | *Pb*ActII | | |
| --- | --- | --- | --- | --- | --- | --- | --- | --- | --- |
|  | d_2-4_ (Å) | b_2_  (Å) | θ  (°) | d_2-4_  (Å) | b_2_  (Å) | θ  (°) | d_2-4_  (Å) | b_2_  (Å) | θ  (°) |
| Ca-ATP | 27.0 | 5.4 | 19.0 | 27.0 | 5.4 | 19.5 | 26.7 | 5.7 | 15.7 |
| Mg-ATP/ADP^*^ | 27.1 | 5.3 | 17.9 | 27.1 | 5.3 | 17.5 | - | - | - |
| Mg-ADP | 27.3 | 5.1 | 20.0 | 26.0 | 5.2 | 21.6 | 25.9 | 5.1 | 18.9 |

|  | *Pf*ActI wt (F) | | | *Dd*Act P109I | | | *Dd*Act P109A | | |
| --- | --- | --- | --- | --- | --- | --- | --- | --- | --- |
|  | d_2-4_  (Å) | b_2_  (Å) | θ  (°) | d_2-4_  (Å) | b_2_  (Å) | θ  (°) | d_2-4_  (Å) | b_2_  (Å) | θ  (°) |
| Ca-ATP | - | - | - | 27.9 | 5.3 | 18.4 | 27.8 | 5.2 | 18.4 |
| Mg-ATP | - | - | - | 28.3 | 5.7 | 18.8 | - | - | - |
| Mg-ADP | 24.2 | 6.2 | 5.1 | - | - | - | 28.2 | 5.1 | 19.4 |

d_2-4_ is the distance between mass centers of SD2 and SD4

b_2_ is the phosphate clamp distance between Cα atoms of Gly16/15 and Asp158/157(defined in ref. 24)

θ is the torsion angle from mass centers of all four subdomains
