# Supplementary figures and images for: Atomic view into Plasmodium actin polymerization, ATP hydrolysis, and fragmentation
Source: PLoS Biol. 2019 Jun 14;17(6):e3000315. doi: 10.1371/journal.pbio.3000315 (PMC6599135; doi:10.1371/journal.pbio.3000315)

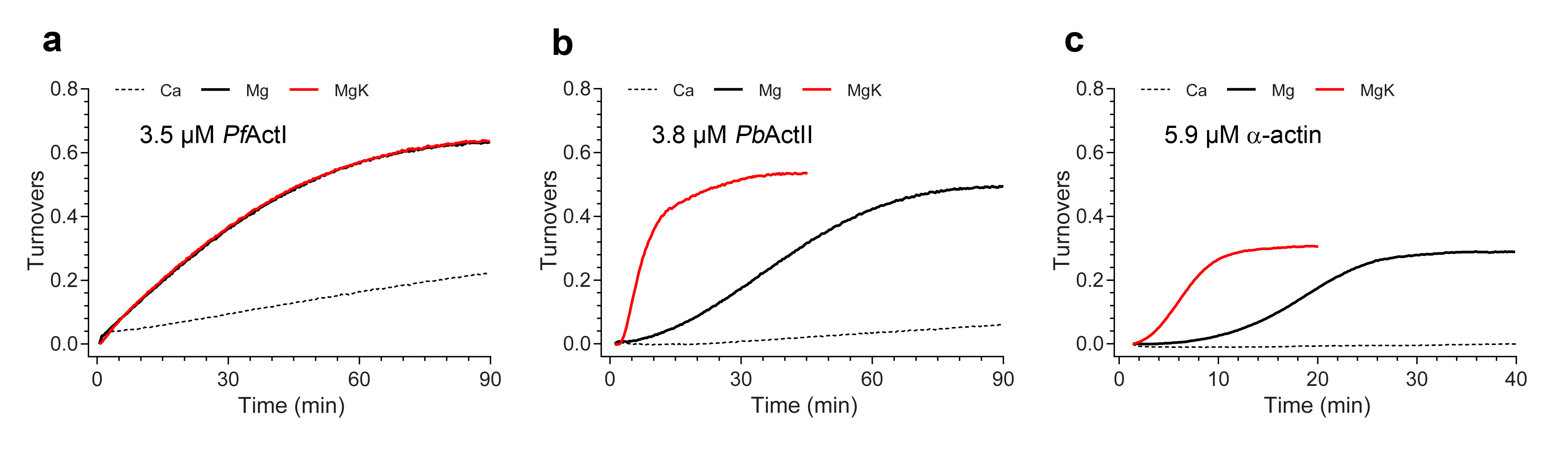

Supplement: S1 Fig — Pi release curves of (A) PfActI at 3.5 μM, (B) PbActII at 3.8 μM, and (C) α-actin at 5.9 μM in Ca-ATP (dashed black line), Mg-ATP (black line) and MgK (red line) conditions. The underlying data for this figure can be found in S4 Data. PbActII, Plasmodium berghei actin II; PfActI, P. falciparum actin I; Pi, inorganic phosphate. (TIF) [file pbio.3000315.s008.tif]

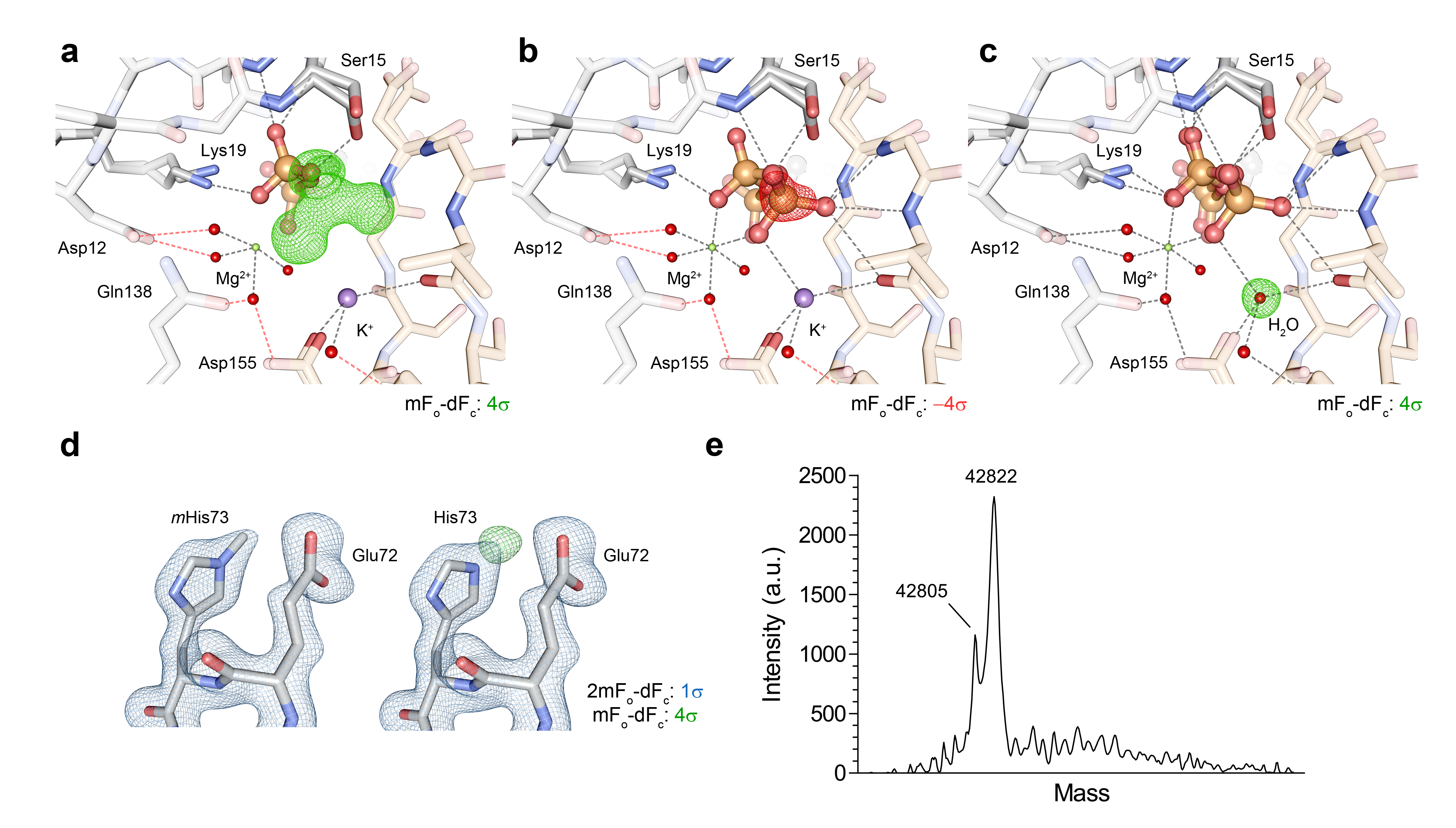

Supplement: S2 Fig — Presence of ATP/ADP mixture and K in the active site of PfActI and the presence of mHis73 in PbActII evidenced by electron density maps and mass spectrometry. Difference density maps (mFo-dFc) at 4 σ of PfActI active site refined with (A) ADP only, (B) ATP only, and (C) K replaced by full-occupancy water. (D) Electron density (2mFo-dFc) and difference density (mFo-dFc) maps around His73 at 1 σ and 4 σ, respectively, with a model refined as methylated and unmethylated His73. (E) Mass spectrum of full-length PbActII expressed in Sf21 insect cells. Spectrum measured by ESI-LCMS from 12 μM PbActII with 1.45% (v/v) TFA. The 42,822-Da peak corresponds to methylated PbActII, whereas the theoretical average molecular weight for unmethylated PbActII is 42,809 Da (Δm = 13 Da). The peak at 42,805 Da is unknown but could result from, e.g., intramolecular disulfides (2 × −2 Da). The underlying data for this figure can be found in S5 Data. ESI-LCMS, electrospray ionization liquid chromatography mass spectrometry; mHis73, methylated His73; PbActII, Plasmodium berghei actin II; PfActI, P. falciparum actin I; Sf21, Spodoptera frugiperda cell line; TFA, trifluoroacetic acid. (TIF) [file pbio.3000315.s009.tif]

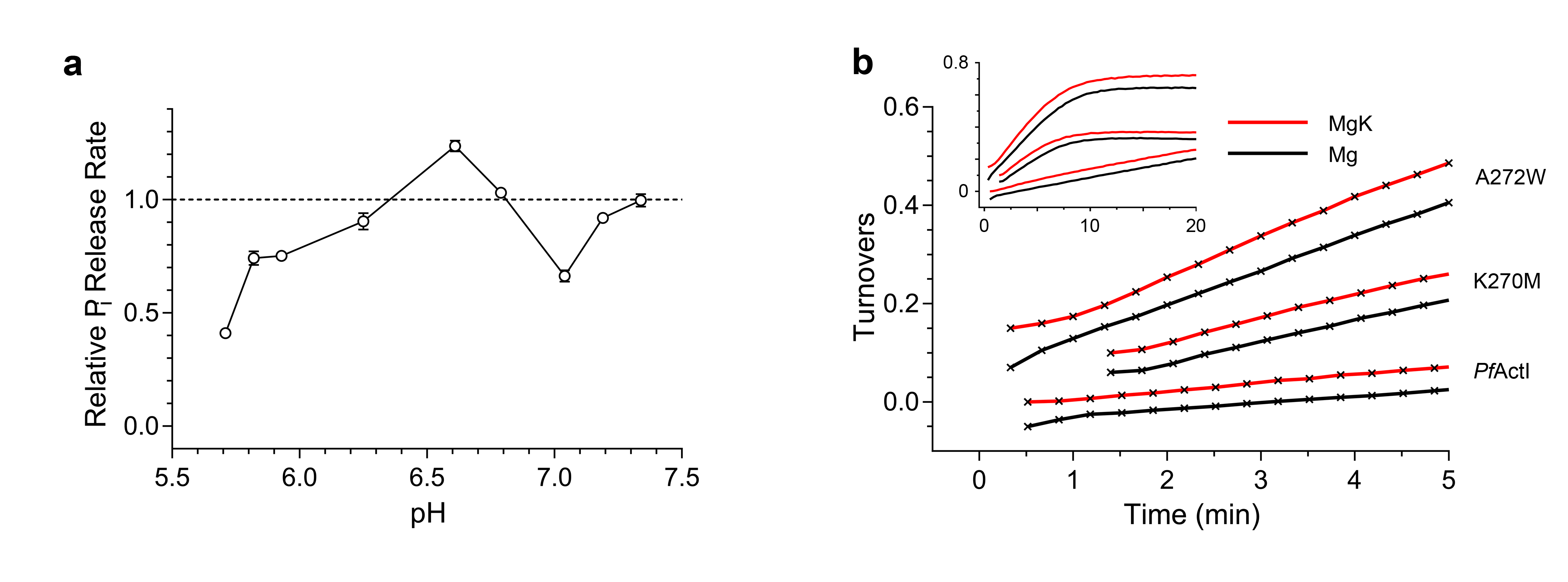

Supplement: S3 Fig — (A) The effect of pH on Pi release by PfActI. The Pi release rate was measured in Mg-ATP conditions at a range of pH values. The rates were normalized relative to the sample at pH 7.34, which corresponds to the standard assay conditions. (B) Delayed phosphate release in mutants K270M and A272W of PfActI. Pi release curves of PfActI wild-type (3.5 μM), K270M (6.4 μM), and A272W (4.3 μM) mutants in Mg-ATP (black lines) and MgK (red lines) conditions. The inset shows the complete curves with the same units as the main plot. The curves have been translated in Y to improve clarity. The underlying data for this figure can be found in S6 Data. PfActI, Plasmodium falciparum actin I; Pi, inorganic phosphate. (TIF) [file pbio.3000315.s010.tif]

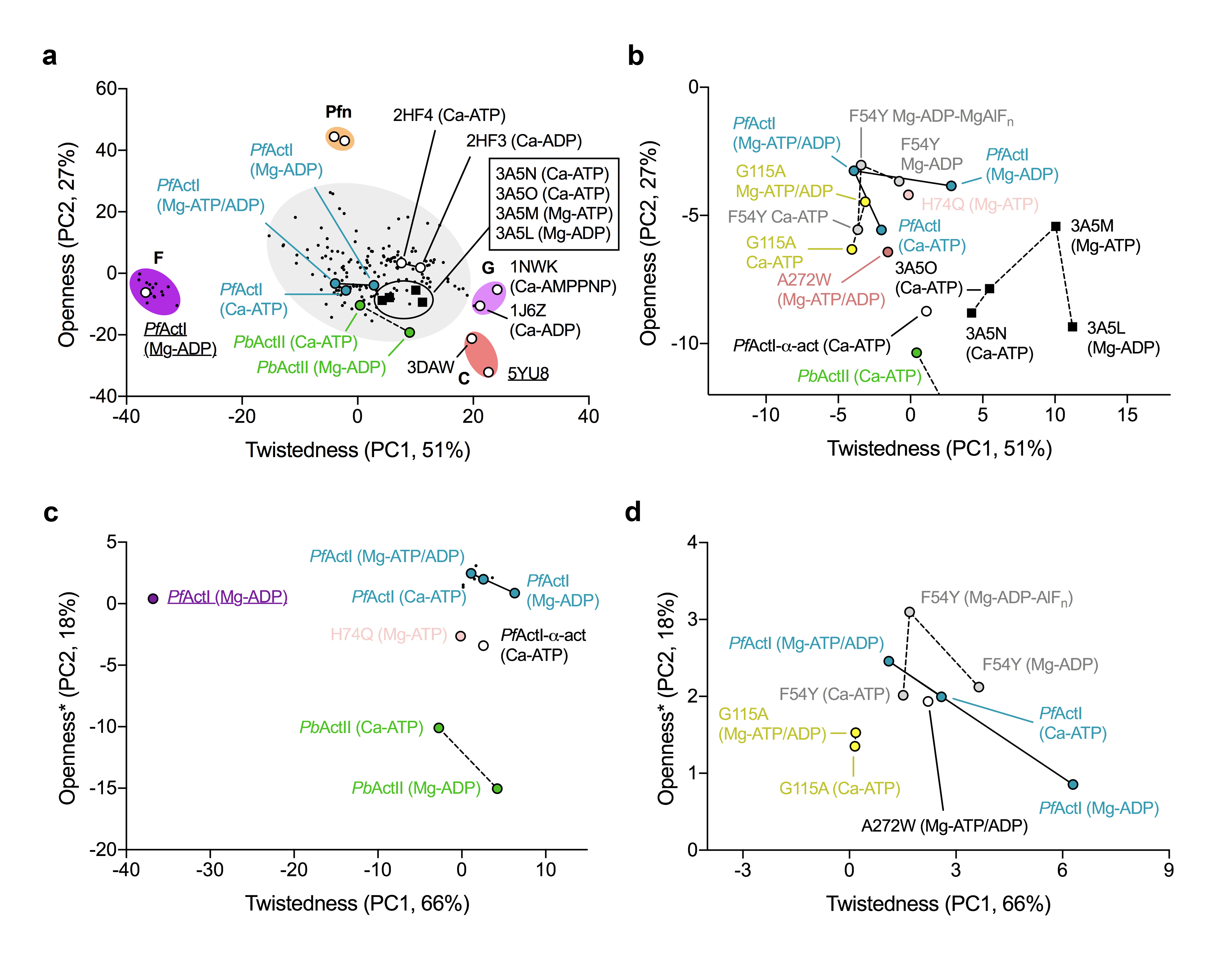

Supplement: S4 Fig — (A) Plot of twistedness (PC1) versus openness (PC2) of the full data set of 147 actin structures (see also S1 Movie). Defined structural groups of filament structures (dark purple), Pfn-bound open structures (orange), free G actin structures (light purple), and ADF/cofilin-bound structures (pink) are indicated with F, Pfn, G, and C, respectively. The large heterogeneous group in the middle is shaded in gray. Structures of interest are indicated with circles or squares and names or PDB identifiers, whereas others are indicated with black dots. (B) Zoomed view of (A) containing the Plasmodium actin structures (excluding PbActII Mg-ADP) as well as 4 mutant D. discoideum actin structures [16] constituting a full set of nucleotide and divalent cation states. (C) PCA of Plasmodium actin structures only, with similar notation as in (A). (D) Zoomed view of (C) containing all relevant PfActI structures excluding the H74Q mutant and the PbActI–α-actin chimera [9]. The lines and dashed lines between the PfActI and PbActII structures indicate the path in the hydrolytic direction (ATP–ATP/ADP–ADP) as appropriate for each set of structures. F structures are underlined, all other structures are of the G form. The underlying data for this figure can be found in S7 Data. ADF, actin depolymerizing factor; C, ADF/cofilin-bound structures; F, filamentous; G, globular; PbActII, Plasmodium berghei actin II; PC, principal component; PCA, principal component analysis; PDB, Protein Data Bank; PfActI, P. falciparum actin I; Pfn, profilin. (TIF) [file pbio.3000315.s011.tif]

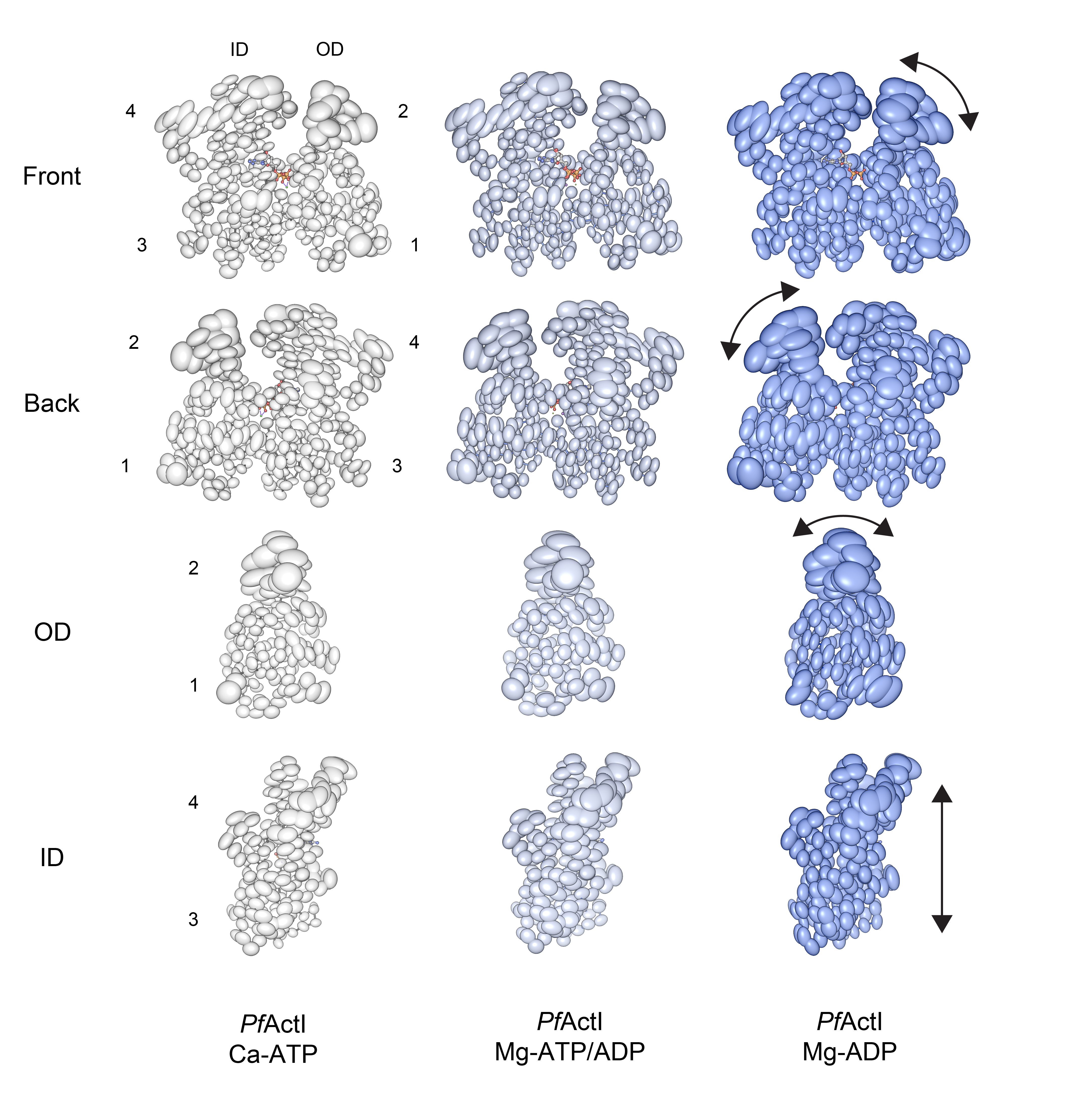

Supplement: S5 Fig — Anisotropic B factors show relative destabilization of SD2 of PfActI during ATP hydrolysis. The size of the ellipsoids is proportional to residue-level B factors and the shape to the direction of anisotropy. Presence of Mg induces an opening of the interdomain cleft and a directional destabilization of SD2 along the rotation of the G–F transition axis (through the mass centers of SD1 and SD3). (Front) View from the side of nucleotide entry (ID on the left, OD on the right). (Back) View from the opposite side of nucleotide entry. (OD) Side view from the OD side with ID faded out. (ID) Side view from the ID side with OD faded out. Numbers 1 through 4 indicate the respective SDs, and the arrows indicate the main direction of anisotropy. F, filamentous; G, globular; ID, inner domain; OD, outer domain; PfActI, Plasmodium falciparum actin I; SD, subdomain. (TIF) [file pbio.3000315.s012.tif]

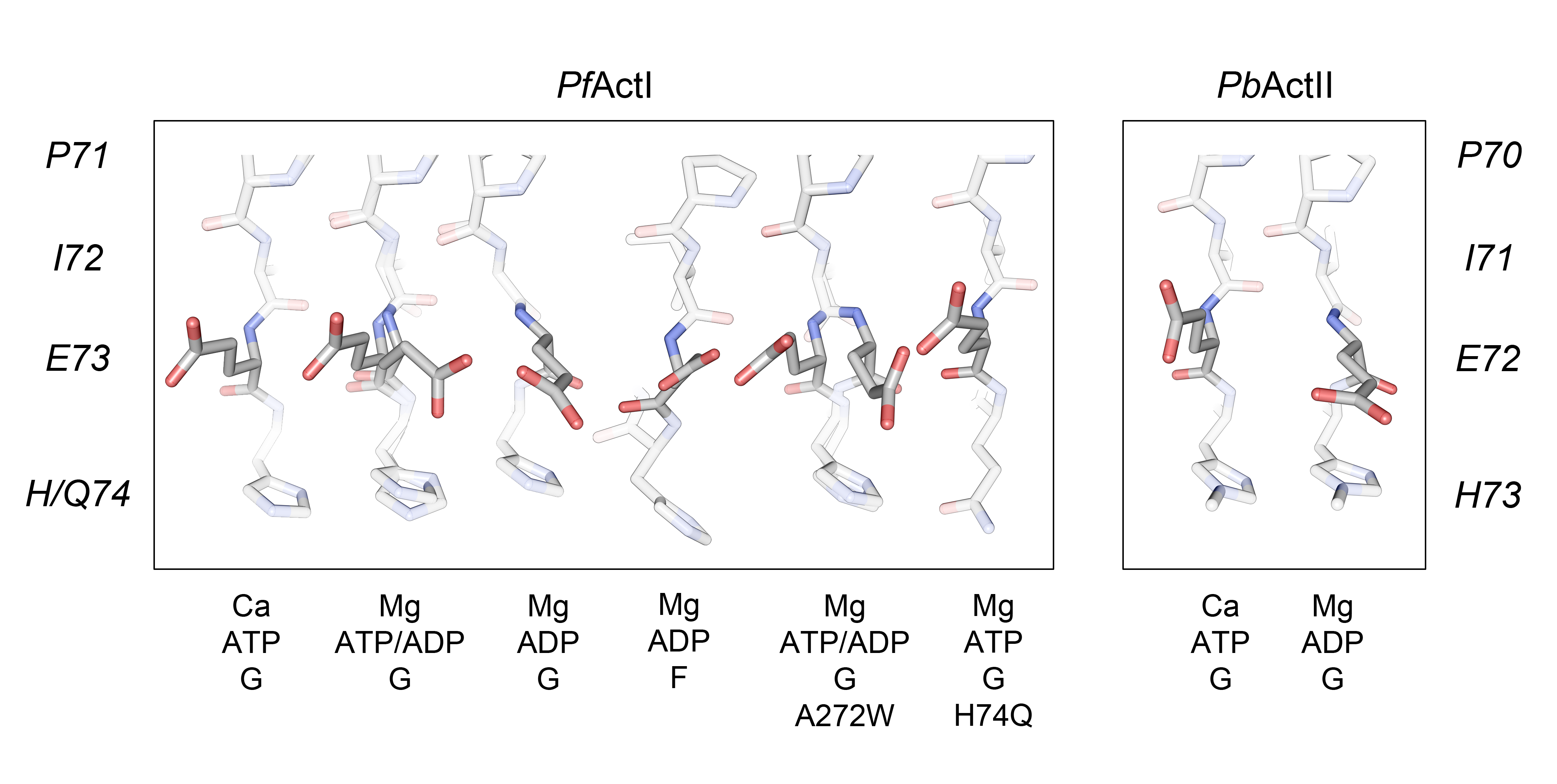

Supplement: S6 Fig — Orientations of the H-loop Glu73-His74 backbone in PfActI wild type and mutants A272W and H74Q as well as those of Glu72-His73 backbone in PbActII. PbActII, Plasmodium berghei actin II; PfActI, P. falciparum actin I. (TIF) [file pbio.3000315.s013.tif]

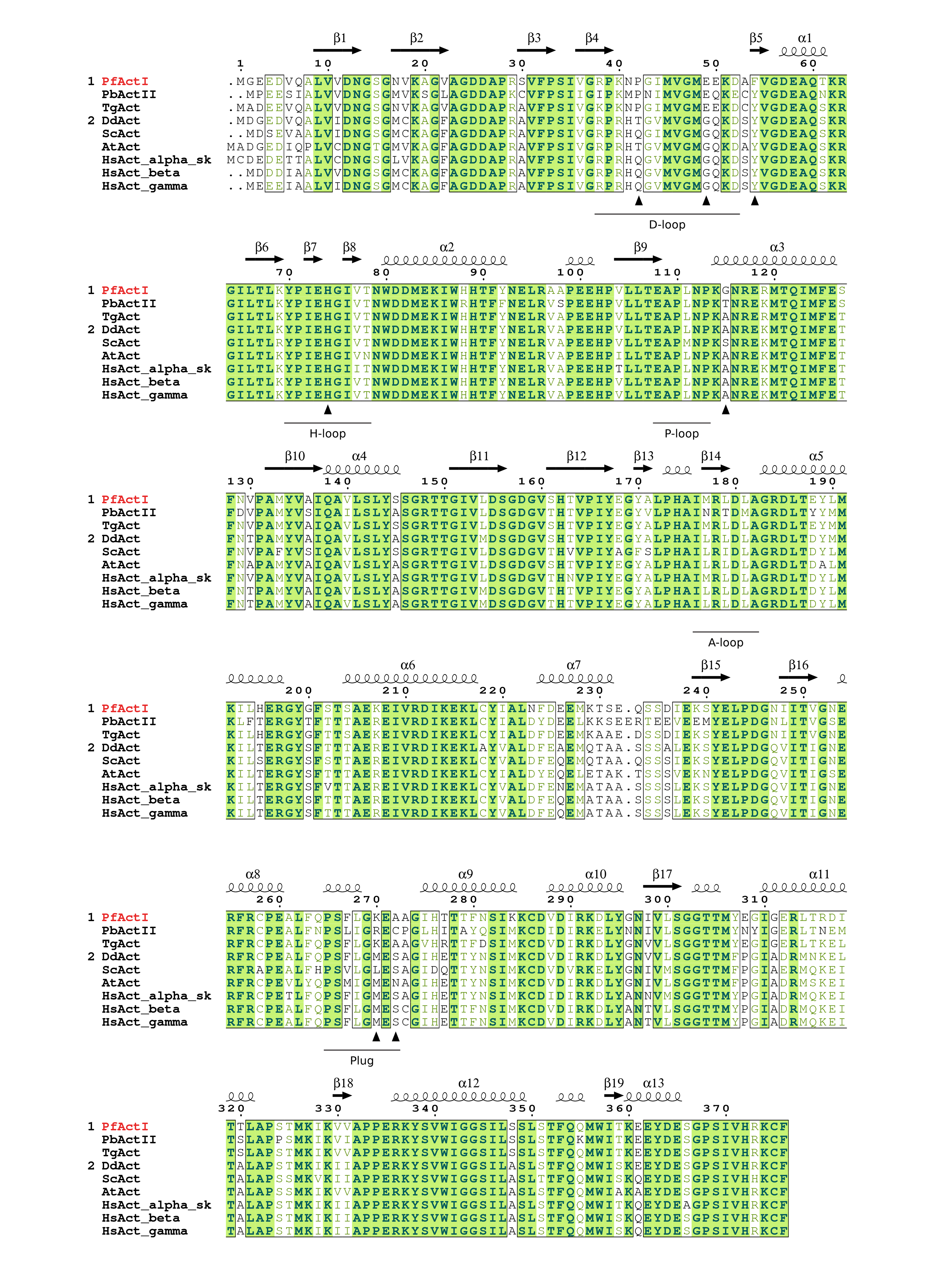

Supplement: S7 Fig — Sequence alignment of selected actin amino acid sequences with secondary structure elements from PfActI [9]. α-helices and β-strands are indicated with helical symbols and arrows as well as Greek letters and numbers, whereas 310 helices are only indicated by helical symbols. The loops and the plug region discussed in the text are highlighted under the sequences. The sequences are divided into 2 groups: (1) apicomplexan actins, including PfActI (Q8I4X0), PbActII (Q4YU79), and TgAct (P53476) and (2) canonical actins from DdAct (P07830), ScAct (P60010), AtAct (P0CJ46), HsAct_alpha_sk (P68133), HsAct_beta (P60709), HsAct_gamma (P63261). Human actins are identical in sequence to corresponding other mammalian actins. Arrowheads indicate mutations studied in this work. The sequence numbering corresponds to that of PfActI. AtAct, Arabidopsis thaliana actin; DdAct, Dictyostelium discoideum actin; HsAct_alpha_sk, Homo sapiens skeletal muscle α actin; HsAct_beta, H. sapiens β actin; HsAct_gamma, H. sapiens γ actin; PbActII, Plasmodium berghei actin II; PfActI, P. falciparum actin I; ScAct, Saccharomyces cerevisiae actin; TgAct, Toxoplasma gondii actin. (TIF) [file pbio.3000315.s014.tif]

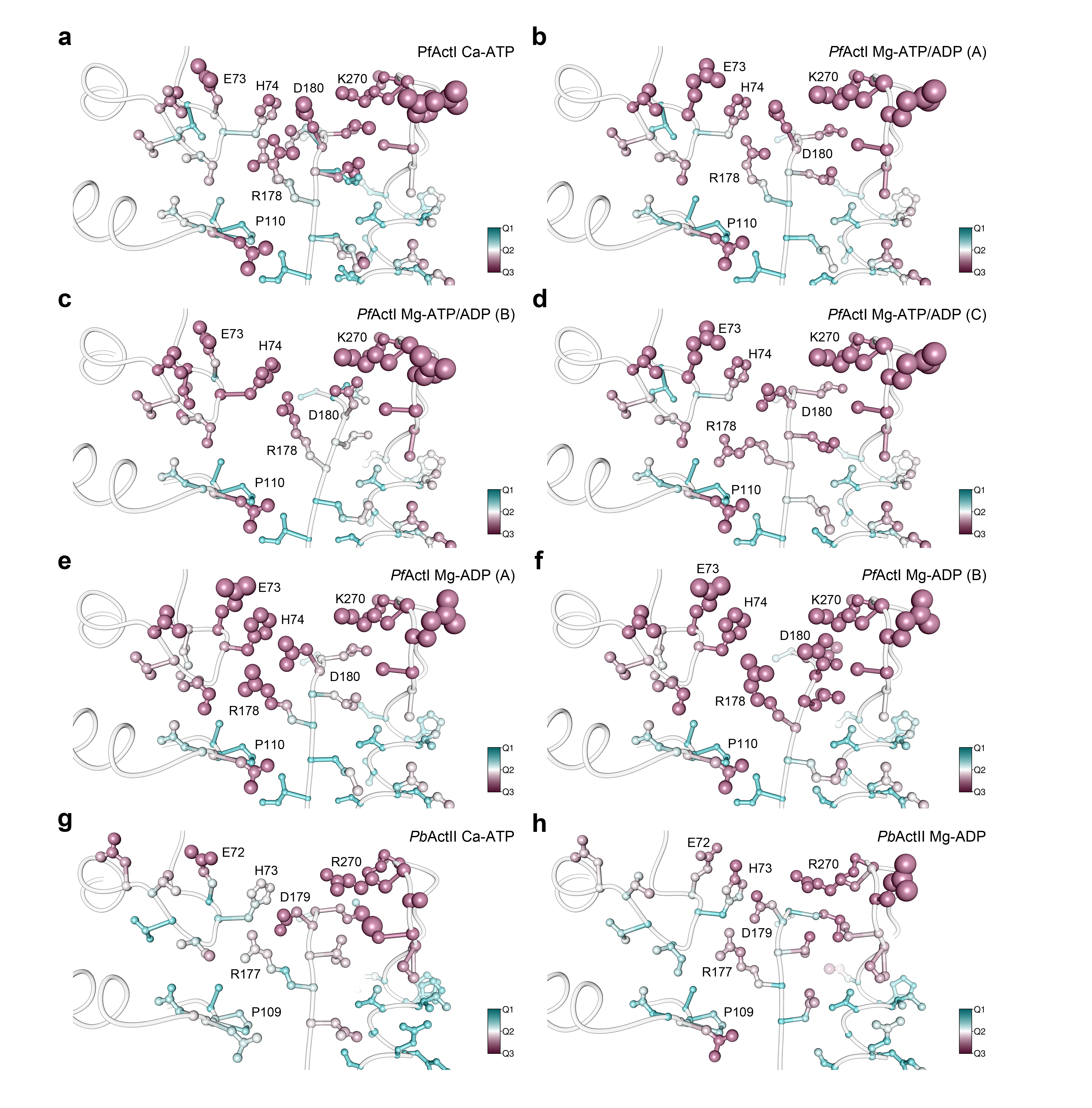

Supplement: S8 Fig — Temperature factors of protein side-chain atoms in the vicinity of the A loop. PfActI in (A) Ca-ATP state (all conformers), Mg-ATP/ADP state conformers A, B, and C (B–D), Mg-ADP state conformers A and B (E, F). PbActII in Ca-ATP state (G) and Mg-ADP state (H). The size of the sphere relates to the maximum and minimum B factors of each structure, whereas the color relates to the first quartile (Q1, dark cyan), median (Q2, white), and third quartile (Q3, maroon) of the total distribution of B factors in each structure. PbActII, Plasmodium berghei actin II; PfActI, P. falciparum actin I. (TIF) [file pbio.3000315.s015.tif]

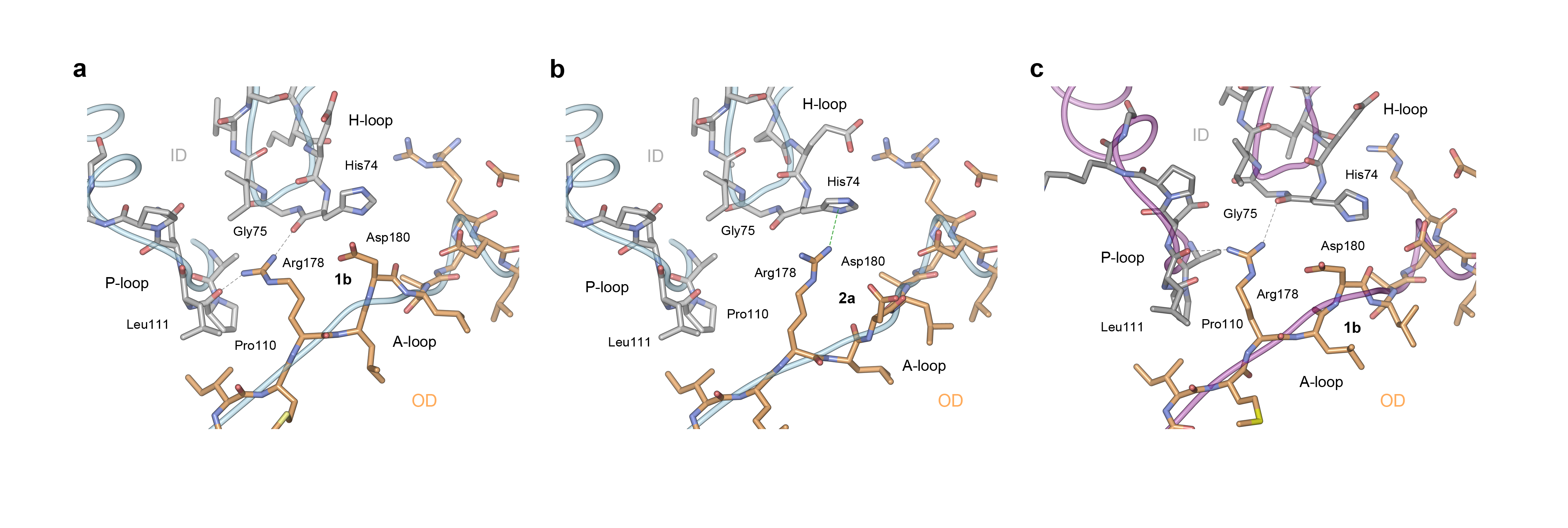

Supplement: S9 Fig — Connections formed by Arg178 between the P loop of SD1 and the H loop of SD2 in the PfActI Mg-ATP/ADP structure. (A) Arg178 forms hydrogen bonds with backbone O of Pro110 in SD1 and His74 in SD2 in conformation 1b. (B) Interaction between Arg178 and SD2 is maintained by the cation-π interaction via His74 in conformation 2a, but the connection to SD1 is lost. (C) Contacts between the ID and OD are preserved in the F-PfActI model that is also in conformation 1b. OD residues are indicated in orange carbon atoms, whereas ID residues are indicated in gray. F, filamentous; ID, inner domain; OD, outer domain; PfActI, Plasmodium falciparum actin I; SD, subdomain. (TIF) [file pbio.3000315.s016.tif]

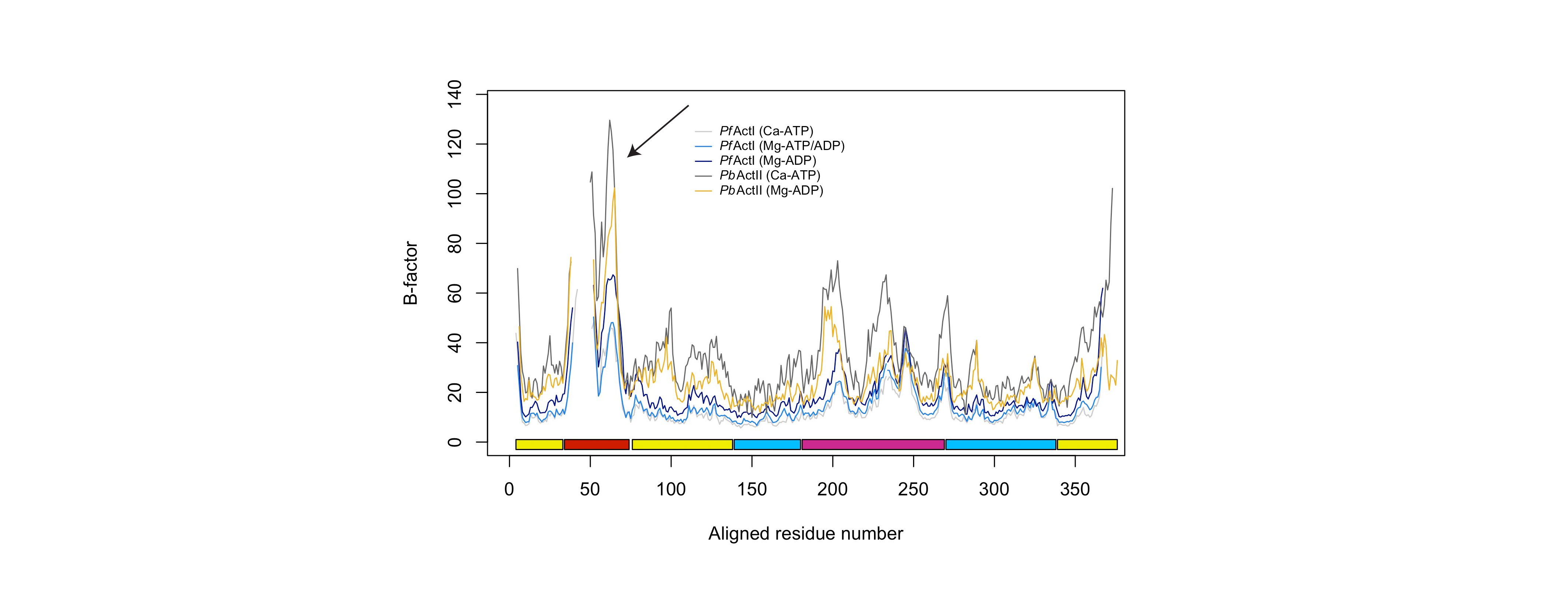

Supplement: S10 Fig — Plot of residue-level B factors of PfActI in (gray line) Ca-ATP, (blue line) Mg-ATP/ADP and (black line) Mg-ADP states as well as PbActII in (brown line) Ca-ATP and (orange line) Mg-ADP states. The SDs are annotated as SD1 (yellow), SD2 (red), SD3 (cyan), and SD4 (magenta). The approximate site of residues 61–66 that are built into weak density is marked by a black arrow. The underlying data for this figure can be found in S8 Data. PbActII, Plasmodium berghei actin II; PfActI, P. falciparum actin I, SD, subdomain. (TIF) [file pbio.3000315.s017.tif]
